# Development of a Synthetic Route to Vonoprazan via Atom Transfer Radical Cyclization

**DOI:** 10.1021/acs.joc.4c02368

**Published:** 2025-02-26

**Authors:** Ken-ichi Ojima, Ryoya Imaizumi, Tatsuya Komori, Toru Nishikawa, Nobuyoshi Doi, Shigenobu Nishiguchi

**Affiliations:** API Process Research I Department, API Business Unit, R&D Division, Towa Pharmaceutical Co., Ltd., Amagasaki Research Incubation Center 3F, 7-1-3, Doi-cho, Amagasaki, Hyogo 660-0083, Japan

## Abstract

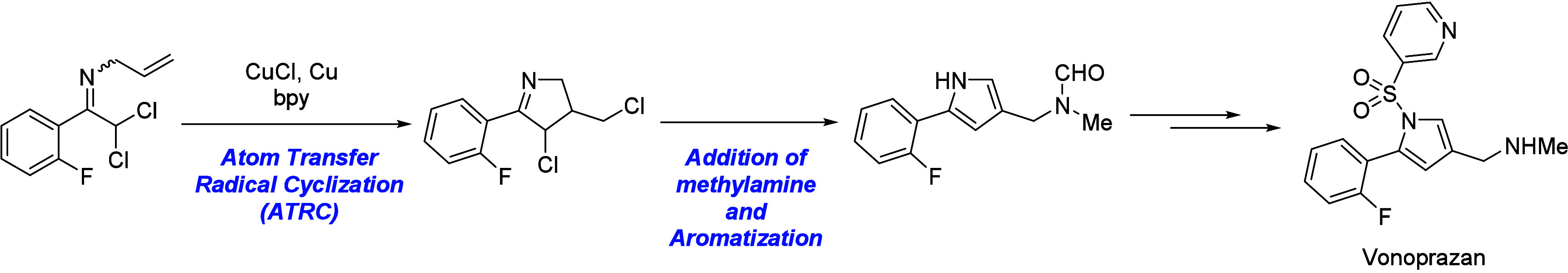

In this Note, a new synthetic route to vonoprazan via atom transfer radical cyclization (ATRC) is described. The pivotal 1,3,5-trisubstituted pyrrole ring of vonoprazan has been accomplished by ATRC, the subsequent aromatization which simultaneously occurred with the introduction of *N*-methylamine moiety into the cyclic imine, and the sulfonylation at the 1-position of pyrrole. Furthermore, our approach enabled the synthesis of vonoprazan on a 0.7 kg scale without the isolation of intermediates throughout the process.

Vonoprazan fumarate developed by Takeda Pharmaceutical Company Limited is one of the proton pump inhibitors (PPIs) and is known as a new class of potassium-competitive acid blocker.^1^ Vonoprazan (**1**) consists of the unique 1,3,5-trisubstituted pyrrole ring system which is characterized by a pyridine-3-sulfonyl group at the 1-position, a methylaminomethyl group at the 3-position, and a 2-fluorophenyl group at the 5-position. Therefore, the efficiency in the construction of this pyrrole ring is crucial, especially in the large scale manufacturing of active pharmaceutical ingredients (APIs). In order to accomplish the synthesis of vonoprazan (**1**), several approaches have been reported,^1−4^ and the synthetic route developed by Takeda is shown in Scheme 1.^2f^ In this synthetic route, 2′-fluoroacetophenone **2** is converted to the dinitrile intermediate **4** via bromination at the α-position of the carbonyl moiety. Subsequently, 3,5-disubstituted pyrrole **5** is obtained by catalytic Pd hydrogenation. After 3 steps that include the reduction of nitrile to aldehyde, sulfonylation, and reductive amination, the synthesis of **1** is accomplished in 40% overall yield. This synthetic route is concise and efficient; however, it requires multiple redox processes and the use of highly toxic transition metals such as Pd and Ni.

**Scheme 1:**
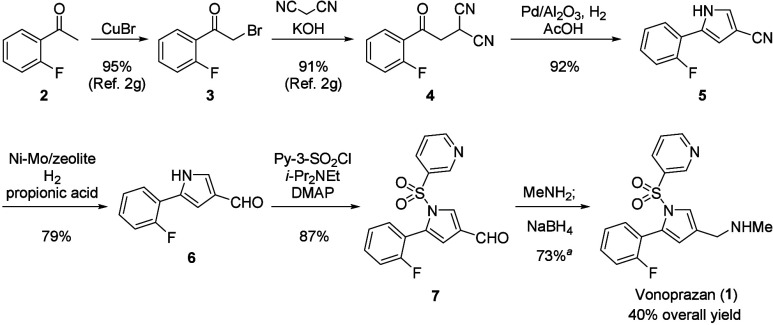
Synthetic Route to Vonoprazan (**1**) Developed by Takeda Pharmaceutical Isolated yield as fumarate.

Atom transfer radical cyclization (ATRC) is a versatile and powerful technique to provide a convenient route for the construction of cyclic frameworks.^5^ However, to our knowledge, there are no reports on ATRC via secondary radical species at the α-position of imine, which affords the multisubstituted pyrrole or dihydropyrrole rings. This fact encouraged us to explore the application of a practical synthesis method for vonoprazan using ATRC. We report here a new synthetic route to **1** that adopts the construction of the 3,5-disubstituted dihydro-2*H*-pyrrole (cyclic imine) via ATRC, the subsequent aromatization which simultaneously occurs with the introduction of *N*-methylamine moiety into the cyclic imine, and the sulfonylation at the 1-position of pyrrole.

In order to develop an elegant and efficient manufacturing route for vonoprazan (**1**), two synthetic routes using halocyclization and atom transfer radical cyclization (ATRC) were explored.

## Part 1: Synthetic Route by Using Halocyclization

**Scheme 2:**
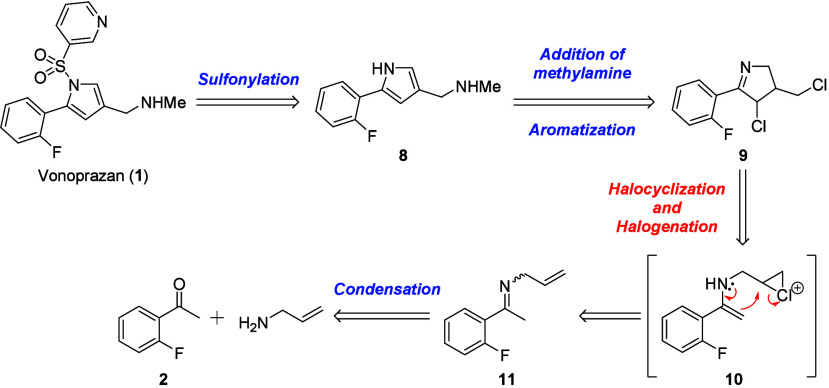
Retrosynthetic Strategy by Using Halocyclization

Initially, we conceived a retrosynthetic strategy for vonoprazan (**1**) by halocyclization (Scheme 2). It was expected that aromatization of dihalogenated cyclic imine **9** would simultaneously occur when the *N*-methylamine moiety was introduced. Regarding the cyclic imine **9**, it would be synthesized by the intramolecular cyclization of **11** via the activation of the terminal olefin with a halogenating reagent.

**Scheme 3:**
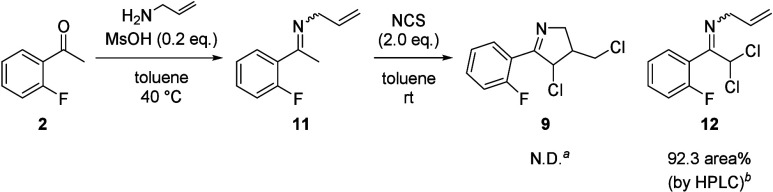
Examination of Halocyclization Not detected by HPLC. The area% of **12** relative to the total area of all peaks in the reaction mixture was determined by HPLC.

With a synthetic strategy in hand, we initiated examinations toward the halocyclization (Scheme 3). The condensation of commercially available 2′-fluoroacetophenone **2** with allylamine proceeded to give the desired allylimine **11**, which was directly used for the next reaction without isolation due to the instability during column chromatography on silica gel. The first investigation for the halocyclization was the treatment of allylimine **11** with 2.0 equiv of *N*-chlorosuccinimide (NCS). However, instead of the cyclic imine **9**, the dichlorinated imine **12** was obtained with a conversion of 92.3 area% by HPLC analysis. Several attempts using other halogenating reagents, such as *N*-bromosuccinimide (NBS) and *N*-iodosuccinimide (NIS), did not afford the cyclic products. We concluded that the tautomerized enamine had a higher affinity for the halogenating reagent than the terminal olefin, and the nucleophilic attack of the enamine was preferred.

***Caution!** Dichlorinated imine **12** was unstable. In our scale-up synthesis of **12** by using 3.86 kg of crude intermediate **11**, a prolonged evaporation of the reaction solution under reduced pressure at 40 °C which required 8 h resulted in the complete degradation of **12**. Therefore, especially in large scale synthesis, dichlorinated imine **12** should be used without evaporation and chromatography on silica gel.*

## Part 2: Synthetic Route by Using ATRC

**Scheme 4:**
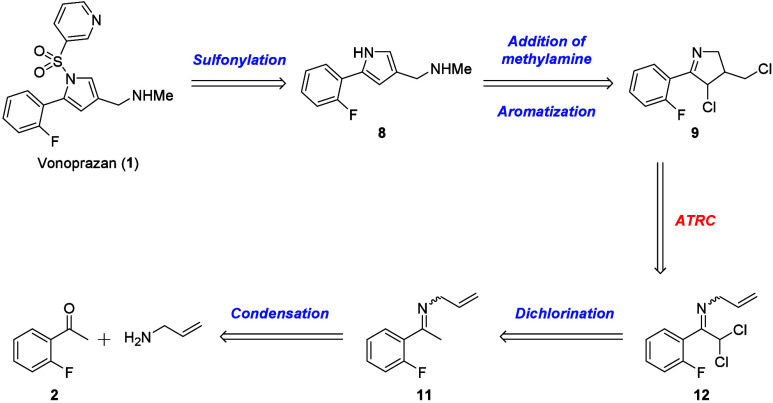
Retrosynthetic Strategy by Using ATRC

While the cyclic imine **9** was not obtained in the approach that used halocyclization as the key reaction, our focus shifted to the use of dichlorinated imine **12** as a substrate for atom transfer radical cyclization (ATRC) (Scheme 4).

**Scheme 5:**
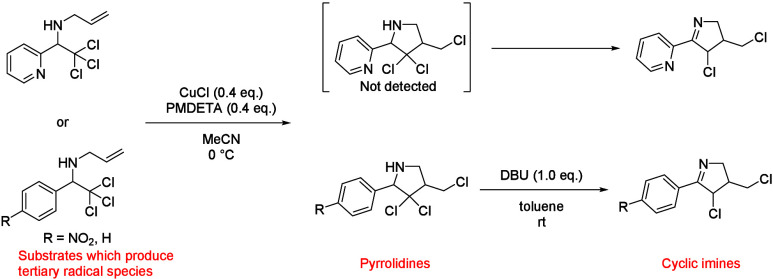
Reported Examples of ATRCs via Tertiary Radical Species

Sadanandan and Gupta et al. have reported ATRCs via various tertiary radical species by using CuCl and pentamethyldiethylenetriamine (PMDETA) as catalyst and ligand, respectively. However, in order to obtain the cyclic imines, DBU-mediated dehydrochlorination and tautomerization of pyrrolidines are necessary, except only when a substrate which has the pyridine moiety is used (Scheme 5).^5b^ In addition, examples using secondary radical species at the α-position of imines have not been reported. Therefore, our challenges were the application of secondary radical species at the α-position of imines for ATRC, and the obtainment of cyclic imine **9** without dehydrochlorination and tautomerization.

The reaction under ATRC conditions that employed CuCl and 2,2′-bipyridine (bpy) as a ligand in toluene at 80 °C was tested (Table 1, entry 1). As expected, the desired cyclic imine **9** was obtained, albeit with low conversion. Further investigations revealed that the solvent choice and the addition of Cu were crucial. The experiments of solvents screening (entries 2–5) provided the best conversion (84.7 area%) by HPLC analysis when the 1,2-dichloroethane (DCE) was used (entry 5). In addition to the improvement of conversion by solvent choice, the required amounts of catalyst and ligand could be reduced by the addition of Cu that was effective in suppressing overoxidation of the copper catalyst^5c^ (entry 6). As discussed so far, ATRC conditions were successfully optimized by using DCE, however this solvent was not favorable for manufacturing of API owing to its high toxicity. After the extensive examinations of solvents (see Table S1 in Supporting Information (SI)), the use of a solvent mixture (toluene and MeCN) yielded a similar conversion (entry 7) comparable to using DCE. Regarding the stereochemistry of **9**, the diastereomer ratios changed little with the different solvents and ligands (*trans*:*cis* = 7:3 to 6:4, see Table S1 in SI). Because the stereochemistry of **9** was destroyed in the subsequent step, optimization studies were not performed.

**Table 1:**
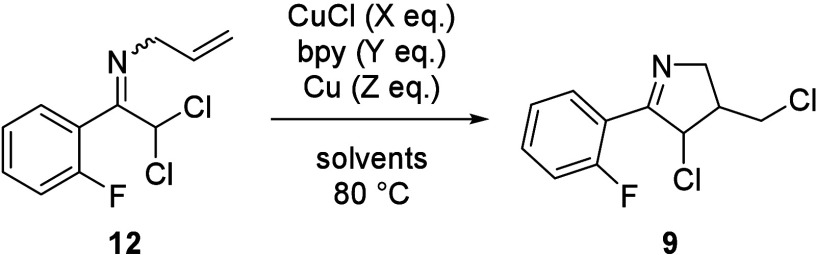
Optimization of Atom Transfer Radical Cyclization

entry	solvents	CuCl (X equiv)	bpy (Y equiv)	Cu (Z equiv)	area% of **9** (HPLC)^a^^,^^c^
1	toluene	0.4	0.8	–	15.0
2	EtOAc^d^	0.4	0.8	–	27.3
3	EtOH^d^	0.4	0.8	–	55.7
4	MeCN	0.4	0.8	–	71.1
5	DCE	0.4	0.8	–	84.7
6	DCE	0.2	0.4	0.2	80.2 (58%^b^^,^^c^)
7	toluene/MeCN (3:1)	0.2	0.4	0.2	84.7

a The area% of **9** relative to the total area of all peaks in the reaction mixture was determined by HPLC.

b Isolated yield.

c After 3 steps conversions from **2**.

d The reaction was performed at 75 °C.

**Scheme 6:**
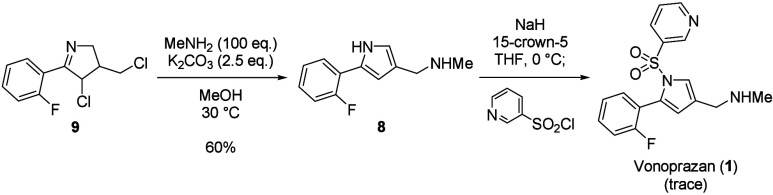
Introduction of Methylamine and Attempted Sulfonylation of Pyrrole

Having optimized conditions for the ATRC to afford cyclic imine **9**, the introduction of methylamine into **9** and the subsequent sulfonylation of pyrrole toward the synthesis of **1** were performed (Scheme 6). The treatment of **9** with an excess amount of methylamine afforded the desired pyrrole **8** in 60% yield. As expected, the aromatization occurred simultaneously. The pyrrole **8** is a known intermediate for the synthesis of **1** and the chemoselective sulfonylation is also documented in a patent.^6^ However, the selective sulfonylation of nitrogen on pyrrole hardly proceeded, and a trace amount of **1** was observed by HPLC analysis.

Considering these results, we decided to utilize the protected methylamines, which could be easily removed after sulfonylation. The suitably protected methylamine derivatives were examined (Table 2), and it turned out that the methylamine protected by the electron-donating Bn group was smoothly introduced to afford the desired pyrrole **13** in 74% yield (entry 1). In addition, protection by the electron-withdrawing Ms group gave pyrrole **14** in 53% yield via deprotonated anion species due to the acidic proton on the sulfonamide (entry 2). Surprisingly, *N*-methylformamide with a weaker acidic proton was also tolerated to afford the corresponding desired product **15** in 33% yield (entry 3). Among these functionalized methylamines, since *N*-methylformamide was the most cost-effective, further optimizations to improve the yield of **15** were investigated (see Table S2 in the SI). Ultimately, the use of KOH in *N*,*N*-dimethylacetamide (DMA) accomplished a 69% yield of **15** (entry 4).

**Table 2:**
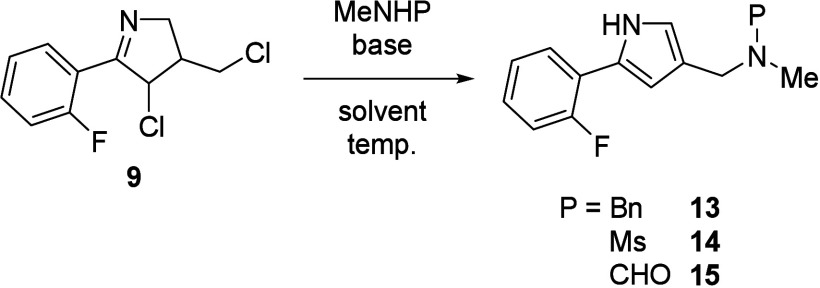
Examinations of Functionalized Methylamines

entry	P	base	solvent	temp (°C)	yield (%)^a^
1	Bn	K_2_CO_3_	MeOH	40	74
2	Ms	K_2_CO_3_	MeOH	35	53
3	CHO	K_2_CO_3_	MeCN	70	33
4	CHO	KOH	DMA	–15	69

a Isolated yield.

**Scheme 7:**
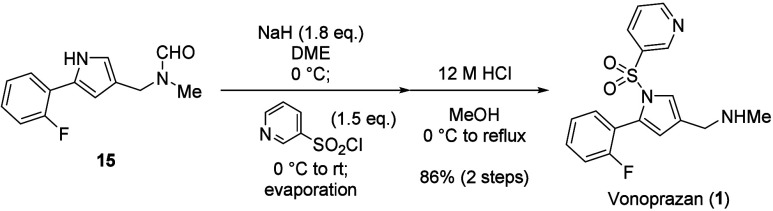
Synthesis of Vonoprazan (**1**) by Sulfonylation and Removal of the Formyl Group

The synthesis of vonoprazan (**1**) was achieved by the remaining requisite transformations (Scheme 7). The sulfonylation of pyrrole proceeded smoothly in 1,2-dimethoxyethane (DME) using NaH as a base. After concentration of the reaction mixture by evaporation, the *N*-formyl group was removed under acidic conditions to afford **1** in 86% yield over 2 steps.

**Scheme 8:**
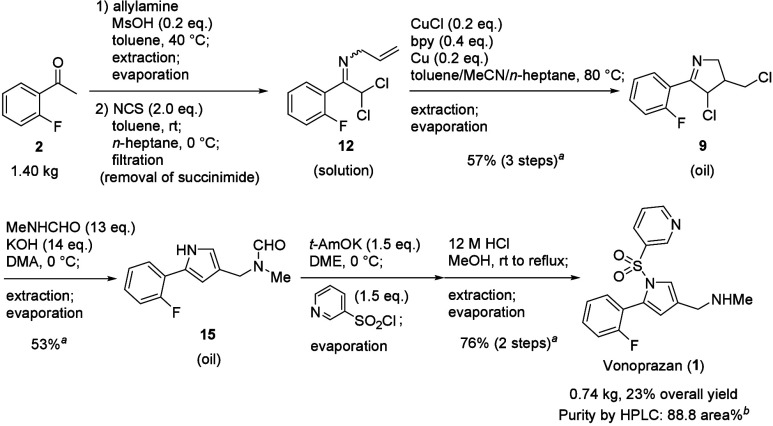
Scale-up Synthesis of Vonoprazan (**1**) Yield was determined by HPLC. The amount of each intermediate or vonoprazan in the crude product was determined by HPLC assay method which used a calibration curve of the purified intermediate or vonoprazan, and then each yield was calculated. The area% of **1** relative to the total area of all peaks in the crude product was determined by HPLC.

Finally, the scale-up synthesis of vonoprazan (**1**) in a telescoping manner was performed (Scheme 8). The use of **2** (1.40 kg) as starting material in our synthetic route consisting of 6 steps without the isolation of intermediates throughout the process successfully afforded **1** (0.74 kg) with a purity of 88.8 area% (HPLC) in 23% overall yield. The purification method of this crude product is also reported in the SI (see Scheme S8).

In conclusion, we have successfully developed a new synthetic route to vonoprazan (**1**) in 6 steps from commercially available 2′-fluoroacetophenone **2** as starting material. The highlights of our synthetic method are as follows. The first is the construction of 3,5-disubstituted dihydro-2*H*-pyrrole (cyclic imine **9**) by using ATRC, which is a first report employing the secondary radical species at the α-position of an imine. The second is that the subsequent aromatization can be simultaneously accomplished when the *N*-methylamine moiety is introduced. We believe that our synthetic method using ATRC is expected to be applied to synthesize various 1,3,5-trisubstituted pyrrole derivatives. Notably, our synthetic method for **1** stands out in terms of redox economy, which requires only one oxidation process (dichlorination by using NCS). Furthermore, we successfully synthesized **1** on a 0.7 kg scale without isolation (telescoping manner) in all steps.

## Data Availability

The data underlying this study are available in the published article and its Supporting Information.

## Supporting Information Available

Optimization of the reaction conditions and experimental details for the synthesis of vonoprazan, NMR spectra, and HPLC chromatograms (PDF)
