# Supplementary material for: Development of a Synthetic Route to Vonoprazan via Atom Transfer Radical Cyclization
Source: J Org Chem. 2025 Feb 26;90(9):3485–8. doi: 10.1021/acs.joc.4c02368 (PMC11894647; doi:10.1021/acs.joc.4c02368)
Supplement: Supplementary file 1 — jo4c02368_si_001.pdf [file jo4c02368_si_001.pdf]

Supporting Information

Development of a Synthetic Route to Vonoprazan via Atom Transfer Radical Cyclization

Ken-ichi Ojima, Ryoya Imaizumi, Tatsuya Komori*, Toru Nishikawa, Nobuyoshi Doi, and Shigenobu Nishiguchi

API Process Research I Department, API Business Unit, R&D Division, Towa Pharmaceutical Co., Ltd., Amagasaki Research Incubation Center 3F, 7-1-3, Doi-cho, Amagasaki, Hyogo 660-0083, Japan

*Corresponding Author: t-komori@towayakuhin.co.jp

Contents

1. General methods S2

2. Optimization of the reaction conditions S3

3. Synthesis of vonoprazan S8

4. Large-scale synthesis of vonoprazan S13

5. NMR spectra S25

1. General methods

All chemicals were purchased from commercial suppliers and used without further purifications unless otherwise mentioned. Small-scale reactions were performed in glass tubes or round-bottomed flasks under a positive pressure of nitrogen unless otherwise mentioned. For kilogram-scale reactions, 20 ~ 100 L-reactors equipped with a mechanical stirrer were used. Anhydrous toluene, EtOAc, *n*-hexane, *n*-heptane, EtOH, MeOH, 1,2-dichloroethene, THF, DMA, 1,2-dimethoxyethane, and *t*-butyl methyl ether were purchased from FUJIFILM Wako Pure Chemical Corporation. Anhydrous MeCN was purchased from KANTO CHEMICAL CO., Inc. TLC analysis was conducted on E. Merck TLC (Silica Gel 60-F_{254} on glass plate). Compounds were visualized by exposure to UV light or by spraying with an acidic staining solution of molybdatophosphoric acid or ethanol solution of ninhydrin followed by heating. HPLC analyses were performed on a Shimadzu Prominence, equipped with ZORBAX Eclipse Plus C18 4.6 x 50 mm (1.8 µm) column manufactured by Agilent Technologies, Inc., CHIRALPAK IB-3 4.6 x 250 mm (3 µm) column manufactured by Daicel Corporation, Inertsil CN-3 4.6 x 150 mm (3 µm) column manufactured by GL Sciences Inc., or ZORBAX Eclipse Plus C18 4.6 x 100 mm (3.5 µm) column manufactured by Agilent Technologies, Inc., respectively. Flash silica gel column chromatography was performed on UNIVERSAL COLUMNS PREMIUM (Yamazen Corporation). Organic solutions were concentrated by rotary evaporation below 40ºC under reduced pressure. Solvent systems in chromatography are specified in v/v. ^{1}H and ^{13}C{^{1}H} NMR spectra were recorded at 295K with a Bruker AVANCE III HD400. Chemical shifts are reported in parts per million (ppm) downfield from Me_4Si. ^{1}H NMR spectra in $CDCl_3$ or DMSO-d_6 were referenced internally to tetramethylsilane as a standard, and ^{13}C NMR spectra to the solvent resonance. Mass spectra were recorded on Thermo Fischer Orbitrap Elite.

2. Optimization of the reaction conditions

Table S1. Atom Transfer Radical Cyclization (ATRC)

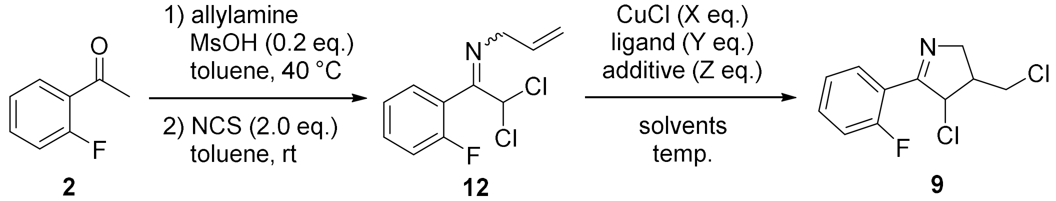

entry	CuCl (X eq.)	ligand (Y eq.)	additive (Z eq.)	solvents	temp. (°C)	area% of **9** (by HPLC)[a, c]	dr[d] (*trans*:*cis*)
1	0.4	bpy (0.8)	-	toluene	80	15.0	64 : 36
2	0.4	bpy (0.8)	-	EtOAc	75	27.3	68 : 32
3	0.4	bpy (0.8)	-	CH_2Cl_2	35	12.9	60 : 40
4	0.4	bpy (0.8)	-	1,4-dioxane	80	49.1	70 : 30
5	0.4	bpy (0.8)	-	EtOH	75	55.7	70 : 30
6	0.4	bpy (0.8)	-	DMAc	80	56.4	69 : 31
7	0.4	bpy (0.8)	-	anisole	80	68.9	64 : 36
8	0.4	bpy (0.8)	-	MeCN	80	71.1	63 : 37
9	0.1	bpy (0.2)	-	MeCN	80	12.4	72 : 28
10	0.4	bpy (0.8)	-	DCE	80	84.7	63 : 37
11	0.4	**L1** (0.8)	-	DCE	80	56.1	68 : 32
12	0.4	**L2** (0.8)	-	DCE	80	62.7	63 : 37
13	0.4	**L3** (0.8)	-	DCE	80	58.3	66 : 34
14	0.4	**L4** (0.4)	-	DCE	80	52.1	61 : 39
15	0.4	bpy (0.8)	MS3A	DCE	80	90.3	63 : 37
16	0.4	bpy (0.8)	PPTS (0.2)	DCE	80	7.2	63 : 37
17	0.4	bpy (0.8)	AcOH (0.2)	DCE	80	17.7	66 : 34
18	0.4	bpy (0.8)	DIPEA (0.2)	DCE	80	73.0	53 : 47
19	0.4	bpy (0.8)	Cu (0.4)	DCE	80	78.8	44 : 56
20	0.2	bpy (0.4)	Cu (0.2)	DCE	80	80.2 (58)[b, c]	60 : 40
21	0.2	bpy (0.4)	Cu (0.2)	toluene/MeCN (3:1)	80	84.7	60 : 40

[a] The area% of **9** relative to the total area of all peaks in the reaction mixture was determined by HPLC.

[b] Isolated yield.

[c] After 3 steps conversions from **2**.

[d] dr was determined by HPLC analysis of the reaction solution.

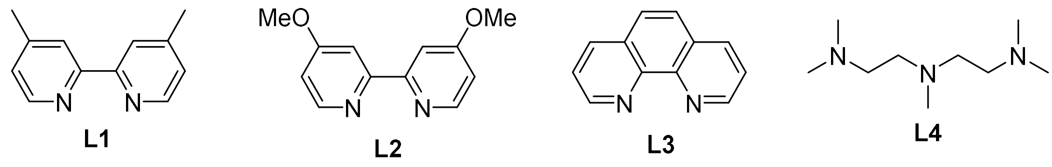

HPLC chromatogram of entry 10 in Table S1[a]

[a] The entry 10 in Table S1 corresponds to entry 5 in Table 1.

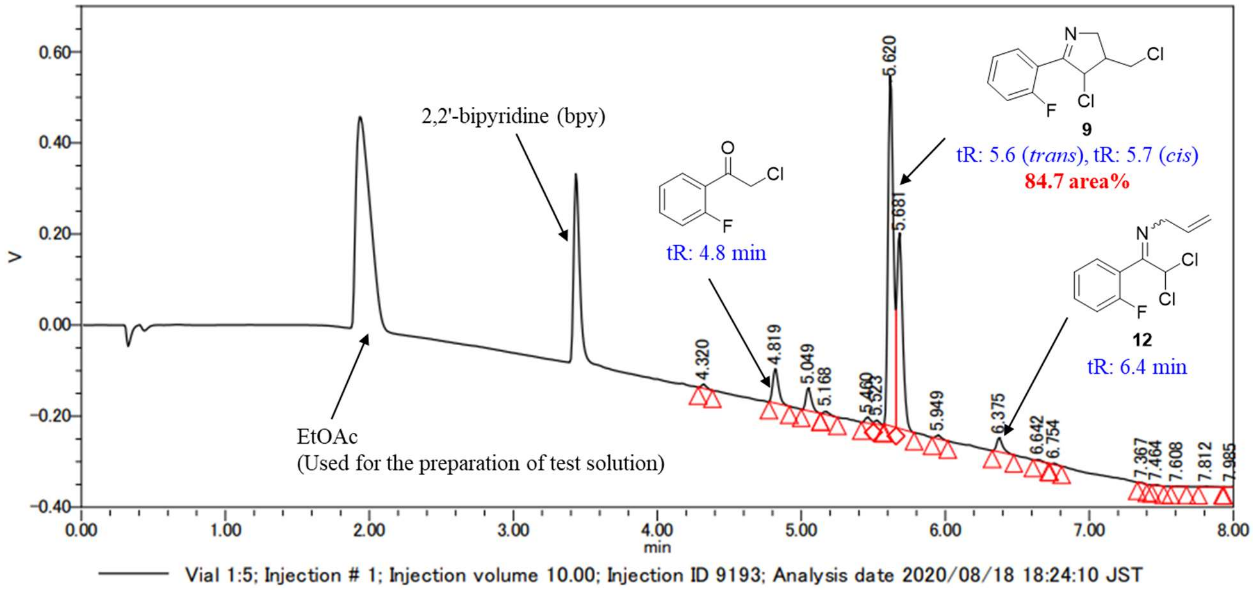

	tR	Area	Area%
1	4.320	21950	0.58
2	4.819	211824	5.63
3	5.049	131470	3.50
4	5.168	18918	0.50
5	5.460	32922	0.88
6	5.523	19359	0.51
7	5.620	2010706	53.47
8	5.681	1175120	31.25
9	5.949	19547	0.52
10	6.375	88252	2.35
11	6.642	8560	0.23
12	6.754	6696	0.18
13	7.367	2577	0.07
14	7.464	2576	0.07
15	7.608	2369	0.06
16	7.812	3346	0.09
17	7.985	887	0.02
18	8.083	3140	0.08

The following HPLC conditions were used. HPLC (C18, gradient method by using A: 10 mM ammonium formate aqueous solution and B: MeCN, flow rate = 1.5 mL/min, l = 210 nm), tR = 5.6 min (compound **9**, *trans*), 5.7 min (compound **9**, *cis*), 6.4 min (compound **12**). The peaks of solvents, 2,2'-bipyridine (bpy), and blank were not integrated.

Other conditions and information are as follows.

Gradient method: The gradient of concentration was controlled by changing the mixing ratios of mobile phase A and B as follows.

Time (min) after injection	A (%)	B (%)
0	90	10
1.00	90	10
6.88	20	80
8.00	20	80

Column: ZORBAX Eclipse Plus C18 4.6 x 50 mm (1.8 μm) manufactured by Agilent Technologies, Inc.

Temperature of column oven: 30 °C

Analysis time: 8 min

Table S2. The introduction of *N*-methylformamide

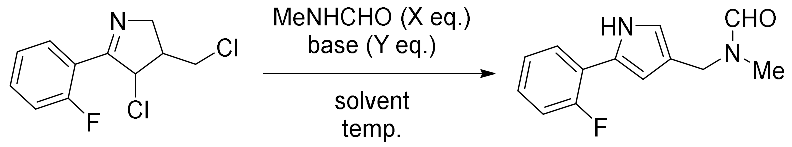

entry	MeNHCHO (X eq.)	base (Y eq.)	solvent	temp. (°C)	area% of **15** (by HPLC)[a]
1	50	K_2CO_3 (10 eq.)	MeCN	70	57.0 (33)[b]
2	10	K_2CO_3 (10 eq.)	MeCN	70	25.5
3	10	K_2CO_3 (10 eq.)	DMA	80	33.6
4	10	NaH (5 eq.)	DMA	0	72.7
5	20	NaH (20 eq.)	DMA	0	82.4
6	20	*t*-BuOK (20 eq.)	DMA	0	81.4
7	20	KOH (20 eq.)	DMA	0	83.2
8	20	KOH (10 eq.)	DMA	0	83.0
9	20	KOH (5 eq.)	DMA	0	82.5
10	20	KOH (10 eq.)	DMA	-15	83.9 (69)[b]
11	10	KOH (10 eq.)	DMA	0	80.2

[a] The area% of **15** relative to the total area of all peaks in the reaction mixture was determined by HPLC.

[b] Isolated yield.

Table S3. The sulfonylation of pyrrole

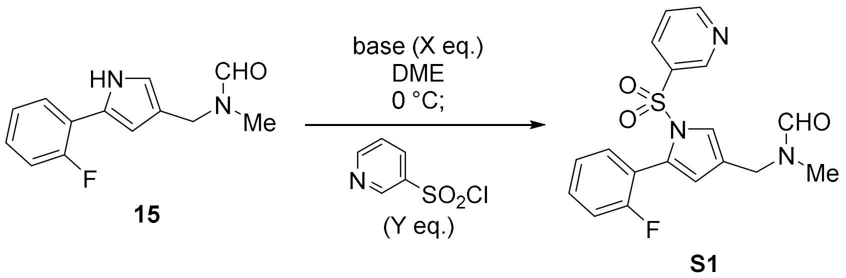

entry	base (X eq.)	Py-3-SO_2Cl (Y eq.)	ratio[a]	
			SM (**15**)	TM (**S1**)
1	NaH (1.8)	1.5	1.7	98.3
2	NaH (1.2)	1.5	4.7	95.3
3	*t*-BuONa (1.2)	1.5	13.2	86.8
4	*t*-BuOK (1.2)	1.5	5.9	94.1
5	*t*-AmONa (1.2)	1.5	2.3	97.7
6	*t*-AmONa (1.1)	1.1	4.6	95.4
7	*t*-AmOK (1.1)	1.1	2.1	97.9

[a] Ratio was determined by HPLC analysis of the reaction solution.

Although the use of NaH afforded the good results in the sulfonylation (entries 1 and 2), NaH is unsuitable for large-scale manufacturing owing to its inflammability. After several investigations, we found that the conditions using *t*-AmOK also resulted in high conversion and used it in the large-scale synthesis of vonoprazan.

3. Synthesis of vonoprazan

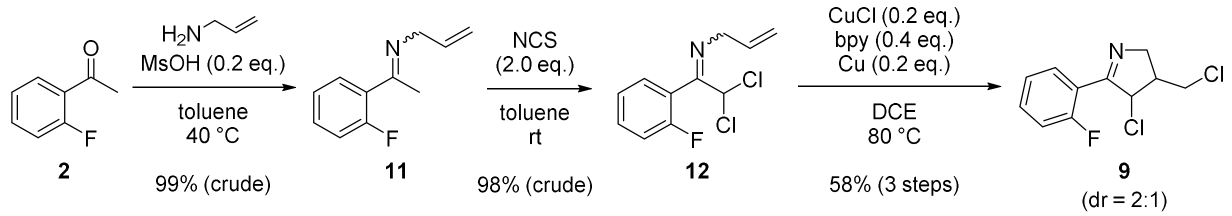

Scheme S1. Synthesis of compound 9

N-[2,2-Dichloro-1-(2-fluorophenyl)ethylidene]-2-propen-1-amine (12)

To a solution of 2'-fluoroacetophenone **2** (100 g, 0.724 mol) in toluene (360 mL) were added allylamine (272 mL, 3.62 mol) and MsOH (9.4 mL, 0.14 mol) at room temperature. The reaction mixture was stirred at 40 °C in an oil bath for 24 h. After cooling to room temperature, 5% aqueous $NaHCO_3$ was added to quench the reaction. Then, the aqueous phase was removed and the organic phase was concentrated under reduced pressure to afford a crude imine **11** (127.4 g, crude, 99%). This crude material was subjected to the next reaction without further purification due to the instability during column chromatography on silica gel.

To a solution of crude imine **11** (823 mg, ca. 4.64 mmol) in toluene (9.3 mL) was added NCS (1.05 g, 7.89 mmol) at room temperature. After stirring for 10 min, additional NCS (0.19 g, 1.4 mmol) was added and the mixture was stirred for 50 min. The reaction mixture was diluted with *n*-hexane and filtered. The filtrate was concentrated under reduced pressure to afford a crude dichlorinated imine **12** (1.12 g, crude, 98%). This crude material was subjected to the next reaction without further purification due to the instability during evaporation and column chromatography on silica gel. ***Caution!*** *Dichlorinated imine* ***12*** *was unstable. In our scale-up synthesis of* ***12*** *by using 3.86 kg of crude intermediate* ***11****, a prolonged evaporation of the reaction solution under reduced pressure at 40 °C which required 8 h resulted in the complete degradation of* ***12****. Therefore, especially in large scale synthesis, dichlorinated imine* ***12*** *should be used without the evaporation and chromatography on silica gel.* **^{1}H NMR** (400 MHz, $CDCl_3$) δ 7.51-7.45 (m, 1H), 7.41-7.37 (m, 1H), 7.27-7.23 (m, 1H), 7.20-7.16 (m, 1H), 6.44 (s, 1H), 5.99-5.90 (m, 1H), 5.16-5.14 (m, 1H), 5.11 (t, J = 1.6 Hz, 1H), 3.88 (br s, 2H); **^{13}C{^{1}H} NMR** (100 MHz, $CDCl_3$) δ 160.8 (d, J = 2.1 Hz), 159.1 (d, J = 246.7 Hz), 134.3, 131.9 (d, J = 8.0 Hz), 130.2 (d, J = 3.6 Hz), 124.3 (d, J = 3.5 Hz), 119.4 (d, J = 18.4 Hz), 116.7, 115.9 (d, J = 21.3 Hz), 73.4, 56.4.

4-Chloro-3-chloromethyl-5-(2-fluorophenyl)-3,4-dihydro-2*H*-pyrrole (9)

To a solution of crude dichlorinated imine **12** (161 mg, ca. 0.653 mmol) in 1,2-dichloroethane (6.5 mL) was added 2,2'-bipyridine (42.5 mg, 0.272 mmol) at room temperature. The pressure inside the flask was reduced, and then nitrogen was introduced to degas the solution (This operation was performed six times). Then, CuCl

(14.4 mg, 0.145 mmol) and Cu powder (9.0 mg, 0.14 mmol) were added and the resulting mixture was stirred at 80 °C in an oil bath for 2.5 h. After cooling to room temperature, the reaction mixture was filtered and concentrated under reduced pressure. The residue was purified by silica gel column chromatography (*n*-hexane-EtOAc = 2:1) to afford cyclic imine **9** (96.1 mg, 0.390 mmol, 58% over 3 steps from **2**) as a brown oil. **^{1}H NMR** (400 MHz, $CDCl_3$, a mixture of diastereomers) δ 7.99 (ddd, *J* = 7.6, 7.6, 1.6 Hz, 0.3H), 7.92 (ddd, *J* = 7.6, 7.6, 2.0 Hz, 0.7H), 7.49-7.43 (m, 1H), 7.27-7.20 (m, 1H), 7.17-7.11 (m, 1H), 5.44 (br d, *J* = 6.4 Hz, 0.3H), 5.30-5.26 (m, 0.7H), 4.37-4.27 (m, 1H), 4.01 (dd, *J* = 17.2, 3.6 Hz, 0.7H), 3.90 (dd, *J* = 10.8, 8.4 Hz, 0.3H), 3.79-3.66 (m, 1.3H), 3.57 (dd, *J* = 11.6, 6.8 Hz, 0.7H), 3.08-2.92 (m, 1H); **^{13}C{^{1}H} NMR** (100 MHz, $CDCl_3$, a mixture of diastereomers) δ 168.9 (d, *J* = 3.0 Hz), 168.2 (d, *J* = 2.6 Hz), 161.2 (d, *J* = 251.6 Hz), 160.9 (d, *J* = 251.0 Hz), 133.0 (d, *J* = 8.8 Hz), 132.8 (d, *J* = 8.7 Hz), 130.9 (d, *J* = 3.1 Hz), 130.8 (d, *J* = 3.0 Hz), 124.63 (d, *J* = 3.4 Hz), 124.55 (d, *J* = 3.4 Hz), 120.0 (d, *J* = 11.8 Hz), 119.8 (d, *J* = 11.1 Hz), 116.5 (d, *J* = 22.6 Hz), 116.3 (d, *J* = 22.5 Hz), 63.8 (d, *J* = 7.2 Hz), 63.5 (d, *J* = 8.6 Hz), 62.3, 61.4, 50.4, 46.7, 44.8, 42.3; **IR** (neat, cm^{-1}) 2955, 2860, 1614, 1487, 1456, 1333, 1300, 1213, 1107, 810, 762; **HRMS** (ESI) m/z: $[M+H]^+$ Calcd for $C_{11}H_{11}NCl_2F$ 246.0247; Found 246.0247.

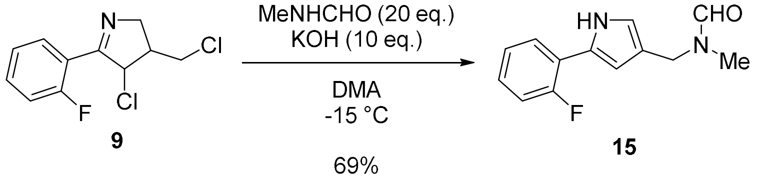

Scheme S2. Synthesis of compound 15

N-[5-(2-Fluorophenyl)-1*H*-pyrrol-3-ylmethyl]-*N*-methylformamide (15)

To a solution of *N*-methylformamide (327 μL, 5.54 mmol) in DMA (0.9 mL) was added KOH (powder, assay: 85%, 184 mg, 2.79 mmol) at room temperature. To this suspension was added a solution of cyclic imine **9** (68.7 mg, 0.279 mmol) in DMA (0.9 mL) at -22~-18 °C. After stirring at -15 °C for an hour, saturated aqueous NH_4Cl was added to quench the reaction and the resulting mixture was extracted with EtOAc three times. The combined organic phase was washed with H_2O and concentrated under reduced pressure. The residue was purified by silica gel column chromatography (*n*-hexane-EtOAc = 1:1) to afford pyrrole **15** (44.6 mg, 0.193 mmol, 69%) as a colorless oil. **^{1}H NMR** (400 MHz, $CDCl_3$, a mixture of rotamers) δ 9.40-9.00 (m, 1H), 8.27 (s, 0.6H), 8.09 (s, 0.4H), 7.61-7.53 (m, 1H), 7.19-7.06 (m, 3H), 6.84 (br dd, *J* = 2.8, 1.6 Hz, 0.4H), 6.80 (br ddd, *J* = 1.6, 1.6, 0.8 Hz, 0.6H), 6.59-6.55 (m, 0.4H), 6.52-6.49 (m, 0.6H), 4.43 (s, 0.8H), 4.31 (s, 1.2H), 2.89 (s, 1.2H), 2.84 (s, 1.8H); **^{13}C{^{1}H} NMR** (100 MHz, $CDCl_3$, a mixture of rotamers) δ 162.6, 162.3, 159.84, 159.79, 157.41, 157.37, 127.80, 127.78, 127.5, 127.4, 127.34, 127.33, 127.28, 127.19, 126.61, 126.59, 126.57, 126.54, 124.75, 124.72, 124.70, 124.66, 120.0, 119.9, 119.8, 119.70, 119.67, 119.4, 118.63, 118.60, 117.91, 117.89, 116.4, 116.3, 116.2, 116.1, 108.2, 108.1, 107.2, 107.1, 46.7, 40.5, 34.0, 29.3; **IR** (neat, cm^{-1}) 3273,

2926, 2864, 1661, 1516, 1487, 1460, 1395, 1217, 814, 758; **HRMS** (ESI) m/z: $[M+Na]^+$ Calcd for $C_{13}H_{13}ON_2FNa$ 255.0904; Found 255.0904.

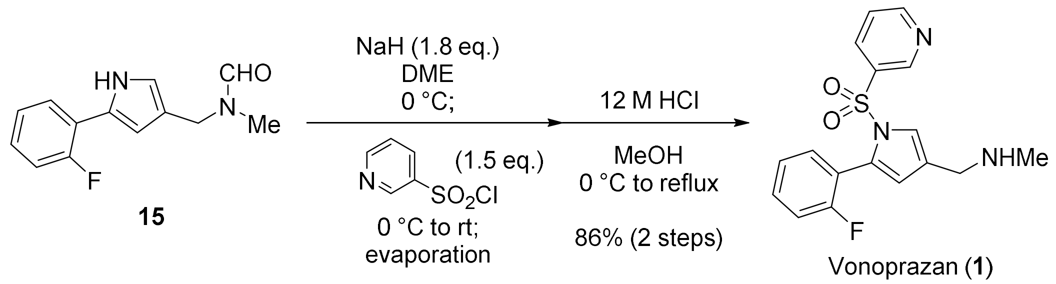

Scheme S3. Synthesis of vonoprazan (1)

1-[5-(2-Fluorophenyl)-1-(pyridin-3-ylsulfonyl)-1*H*-pyrrol-3-yl]-*N*-methylmethanamine (1)

To a solution of pyrrole **15** (13.5 mg, 58.1 μmol) in 1,2-dimethoxyethane (0.12 mL) was added NaH (assay: 60%, 4.2 mg, 0.11 mmol) at 0 °C. After stirring for 10 min, pyridine-3-sulfonylchloride (10.6 μL, 87.2 μmol) was added at 0 °C. The mixture was stirred at room temperature for an hour, and then concentrated under reduced pressure. Next, the residue was diluted with MeOH (0.5 mL). To the mixture was added 12 M HCl (0.1 mL) at 0 °C and the resulting mixture was refluxed in an oil bath for 2.5 h. After cooling to 0 °C, the reaction was quenched with saturated aqueous Na_2CO_3. The mixture was extracted with EtOAc three times and the combined organic phase was concentrated under reduced pressure. The residue was purified by amino silica gel column chromatography (EtOAc) to afford vonoprazan **1** (17.2 mg, 49.8 μmol, 86% over 2 steps from **15**) as a colorless oil. **^{1}H NMR** (400 MHz, $CDCl_3$) δ 8.75 (dd, *J* = 4.8, 1.6 Hz, 1H), 8.61 (dd, *J* = 2.4, 0.4 Hz, 1H), 7.70 (m, 1H), 7.44-7.36 (m, 2H), 7.35-7.30 (m, 1H), 7.20-7.11 (m, 2H), 7.07-7.01 (m, 1H), 6.26 (d, *J* = 2.0 Hz, 1H), 3.61 (s, 2H), 2.45 (s, 3H); **^{13}C{^{1}H} NMR** (100 MHz, $CDCl_3$) δ 160.7 (d, *J* = 247.8 Hz), 154.1, 147.8, 135.1, 134.6, 133.1 (d, *J* = 1.6 Hz), 131.1 (d, *J* = 8.2 Hz), 128.9, 127.6, 123.44, 123.37 (d, *J* = 3.8 Hz), 121.2, 119.1 (d, *J* = 15.3 Hz), 118.1, 115.3 (d, *J* = 21.8 Hz), 48.0, 36.1; **IR** (neat, cm^{-1}) 2849, 2793, 1574, 1470, 1418, 1375, 1227, 1182, 1107, 1072, 820, 762, 750, 700, 627; **HRMS** (ESI) m/z: $[M+H]^+$ Calcd for $C_{17}H_{17}O_2N_3FS$ 346.1020; Found 346.1017.

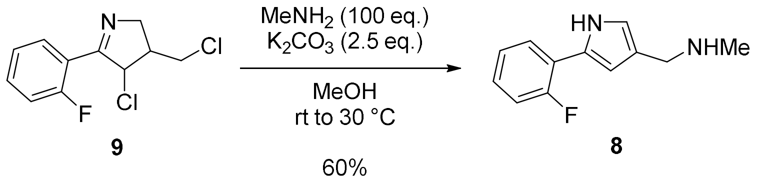

Scheme S4. Synthesis of compound 8

1-[5-(2-Fluorophenyl)-1*H*-pyrrol-3-yl]-*N*-methylmethanamine (8)

To a flask were added cyclic imine **9** (37.2 mg, 0.151 mmol), K_2CO_3 (52 mg, 0.38 mmol), and $MeNH_2$ in MeOH (9.8 M, 1.54 mL, 15.1 mmol) at room temperature, successively. The resulting suspension was stirred at 30 °C in an oil bath for 5 days. Then, the reaction mixture was concentrated under reduced pressure. The residue was purified by amino silica gel column chromatography (EtOAc) to afford pyrrole **8** (18.6 mg, 91.1 μmol, 60%) as a white solid. **^{1}H NMR** (400 MHz, $CDCl_3$) δ 8.95 (br s, 1H), 7.62-7.54 (m, 1H), 7.16-7.04 (m, 3H), 6.81 (br s, 1H), 6.62 (br dd, J = 2.4, 2.0 Hz, 1H), 3.68 (s, 2H), 2.49 (s, 3H); **^{13}C{^{1}H} NMR** (100 MHz, $CDCl_3$) δ 158.6 (d, J = 242.4 Hz), 127.0 (d, J = 2.2 Hz), 126.9 (d, J = 8.7 Hz), 126.5 (d, J = 4.5 Hz), 124.6 (d, J = 3.1 Hz), 123.9, 120.2 (d, J = 11.3 Hz), 117.5 (d, J = 2.8 Hz), 116.2 (d, J = 23.1 Hz), 107.7 (d, J = 2.1 Hz), 48.6, 36.1; **IR** (neat, cm^{-1}) 2941, 2853, 2795, 1614, 1574, 1516, 1487, 1462, 1435, 1217, 1192, 1136, 1105, 1053, 986, 935, 812, 754; **HRMS** (ESI) m/z: $[M+H]^+$ Calcd for $C_{12}H_{14}N_2F$ 205.1136; Found 205.1131.

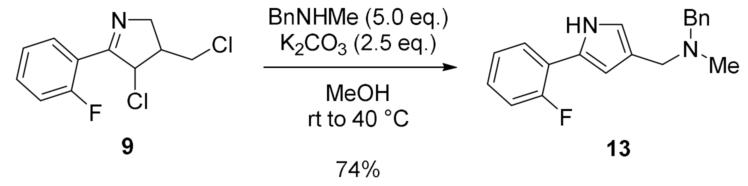

Scheme S5. Synthesis of compound 13

1-[5-(2-Fluorophenyl)-1*H*-pyrrol-3-yl]-*N*-methyl-*N*-methylbenzenemethanamine (13)

To a solution of cyclic imine **9** (39.6 mg, 0.161 mmol) in MeOH (0.8 mL) were added K_2CO_3 (56 mg, 0.41 mmol) and *N*-methylbenzylamine (104 μL, 0.807 mmol) at room temperature. The resulting suspension was stirred at 40 °C in an oil bath for 29 h. Then, the reaction mixture was filtered and concentrated under reduced pressure. The residue was purified by silica gel column chromatography (*n*-hexane-EtOAc = 1:1) to afford pyrrole **13** (34.9 mg, 0.119 mmol, 74%) as a colorless oil. **^{1}H NMR** (400 MHz, $CDCl_3$) δ 8.90 (br s, 1H), 7.61-7.55 (m, 1H), 7.37-7.27 (m, 4H), 7.26-7.19 (m, 1H), 7.13-7.02 (m, 3H), 6.77 (br dd, J = 2.4, 1.6 Hz, 1H), 6.64 (br dd, J = 2.4, 2.0 Hz, 1H), 3.52 (s, 2H), 3.50 (s, 2H), 2.23 (s, 3H); **^{13}C{^{1}H} NMR** (100 MHz, $CDCl_3$) δ 158.6 (d, J = 242.6 Hz), 139.5, 129.2, 128.3, 126.93 (d, J = 8.6 Hz), 126.91, 126.6 (d, J = 4.7 Hz), 124.7 (d, J = 3.0 Hz), 121.9, 120.3 (d, J = 11.2 Hz), 118.7 (d, J = 2.7 Hz), 116.4, 116.1, 108.9 (d, J = 2.1 Hz), 61.3, 54.2, 42.2; **IR** (neat, cm^{-1}) 3472, 3061, 3028, 2940, 2837, 2785, 1516, 1495, 1485, 1456, 1366, 1215, 1125, 1103, 1024,

984, 937, 810, 754, 700; **HRMS** (ESI) m/z: $[M+H]^+$ Calcd for $C_{19}H_{20}N_2F$ 295.1605; Found 295.1602.

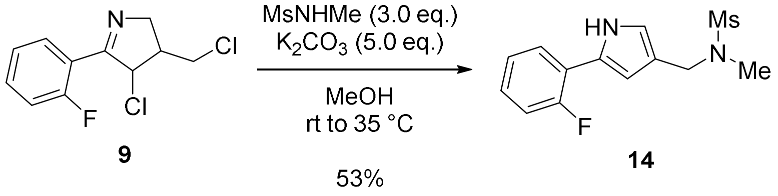

Scheme S6. Synthesis of compound 14

***N*-[5-(2-Fluorophenyl)-1*H*-pyrrol-3-ylmethyl]-*N*-methylmethanesulfonamide (14)**

To a solution of cyclic imine **9** (1.92 g, 7.78 mmol) in MeOH (9.6 mL) were added K_2CO_3 (5.38 g, 38.9 mmol) and *N*-methylmethanesulfonamide (2.0 mL, 23 mmol) at room temperature. The resulting suspension was stirred at 35 °C in an oil bath for 21 h. Then, the reaction mixture was concentrated under reduced pressure. The residue was diluted with EtOAc and H_2O, and extracted with EtOAc three times. The combined organic phase was concentrated under reduced pressure. The residue was purified by silica gel column chromatography (*n*-hexane-EtOAc = 1:1) to afford pyrrole **14** (1.16 g, 4.11 mmol, 53%) as a colorless oil. **^{1}H NMR** (400 MHz, $CDCl_3$) δ 9.03 (br s, 1H), 7.62-7.56 (m, 1H), 7.20-7.07 (m, 3H), 6.89 (br dd, J = 2.4, 1.6 Hz, 1H), 6.64 (br dd, J = 2.4, 2.0 Hz, 1H), 4.28 (s, 2H), 2.85 (s, 3H), 2.74 (s, 3H); **^{13}C{^{1}H} NMR** (100 MHz, $CDCl_3$) δ 158.6 (d, J = 242.8 Hz), 127.6 (d, J = 2.0 Hz), 127.5 (d, J = 8.7 Hz), 126.6 (d, J = 4.4 Hz), 124.8 (d, J = 3.2 Hz), 119.7 (d, J = 11.0 Hz), 118.9 (d, J = 2.7 Hz), 118.6, 116.3 (d, J = 22.7 Hz), 108.2 (d, J = 2.3 Hz), 46.9, 36.2, 34.2; **IR** (neat, cm^{-1}) 3387, 1516, 1487, 1460, 1323, 1150, 970, 912, 758, 517; **HRMS** (ESI) m/z: $[M+Na]^+$ Calcd for $C_{13}H_{15}O_2N_2FNaS$ 305.0730; Found 305.0732.

4. Large-scale synthesis of vonoprazan

4.1. Synthesis of crude vonoprazan

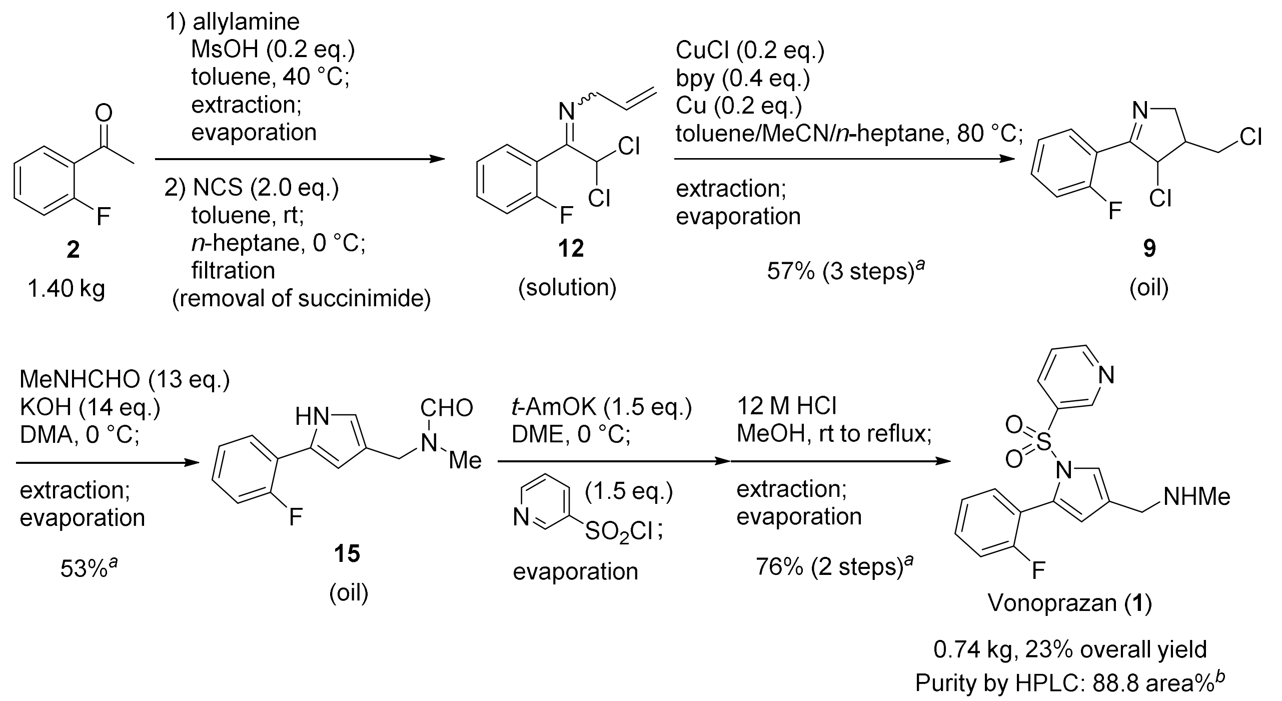

[a]Yield was determined by HPLC. The amount of each intermediate or vonoprazan in the crude product was determined by HPLC assay method which used a calibration curve of the purified intermediate or vonoprazan, and then each yield was calculated. [b]The area% of **1** relative to the total area of all peaks in the crude product was determined by HPLC.

Scheme S7. Large-scale synthesis of vonoprazan (1)

To a solution of 2'-fluoroacetophenone **2** (1.40 kg, 10.1 mol) in toluene (4.46 kg) were added allylamine (2.89 kg, 50.7 mol) and MsOH (0.190 kg, 2.03 mol) at room temperature. The reaction mixture was stirred at 40 °C in a hot water bath for 27 h. After cooling to room temperature, 5% aqueous $NaHCO_3$ (5.17 kg) was added to quench the reaction and the resulting mixture was diluted with toluene (7.79 kg). After the aqueous phase was separated, the organic phase was concentrated under reduced pressure to afford a crude imine **11** (1.98 kg). To a solution of crude imine **11** (1.96 kg) in toluene (14.8 kg) was added NCS (purity: 99.1%, 2.59 kg, 19.2 mol) over the course of 38 min at 20-38 °C (NCS was added in three portions in the order of 1.0, 0.5, and 0.5 equivalent). The reaction mixture was stirred at 22-27 °C for 4 h. Then, the reaction mixture was diluted with *n*-heptane (10.3 kg) and stirred at 0-5 °C for 18 h. The resulting suspension was filtered to give dichlorinated imine **12** in toluene/*n*-heptane solution. ***Caution!*** *Dichlorinated imine* ***12*** *was unstable. In our scale-up synthesis of* ***12*** *by using 3.86 kg of crude intermediate* ***11****, a prolonged evaporation of the reaction solution under reduced pressure at 40 °C which required 8 h resulted in the complete degradation of* ***12****. Therefore, especially in large scale synthesis, dichlorinated imine* ***12*** *should be used without the evaporation and*

chromatography on silica gel.

To a solution of crude dichlorinated imine **12** were added 2,2'-bipyridine (600 g, 3.84 mol), toluene (37.8 kg), and MeCN (14.9 kg) at room temperature. The pressure inside the reactor was reduced, and then nitrogen was introduced to degas the solution (This operation was performed six times). After degassing, to the mixture were added CuCl (190 g, 1.92 mol) and Cu powder (120 g, 1.89 mol), and the resulting mixture was stirred at 80 °C in an oil bath for 3 h. Then, the reaction mixture was concentrated to approximately 47 L under reduced pressure. The mixture was filtered and the residue was washed with toluene (4.10 kg). To the combined filtrate were added KC FLOCK® (1.18 kg) and 0.5 M HCl (8.43 kg) at 20-25 °C. After stirring for 40 min, the mixture was filtered and the residue was washed with toluene (4.12 kg). Then, the aqueous phase of the filtrate was separated and the organic phase was washed with 10% w/w aqueous NaCl solution (5.03 kg). After the aqueous phase was separated, the organic phase was concentrated under reduced pressure. The residue was filtered through a pad of silica gel (4.72 kg, eluent: *n*-heptane/EtOAc = 43.6 kg/19.2 kg) and the filtrate was concentrated under reduced pressure to afford a crude cyclic imine **9** (1.70 kg, the weight which was determined by HPLC assay method: 1.41 kg, 5.73 mol, 57% over 3 steps from **2**).

To a suspension of KOH (powder, assay: 94.0%, 4.40 kg, 73.7 mol) in DMA (11.2 kg) was added *N*-methylformamide (4.05 kg, 68.6 mol) at 0-10 °C. Next, to this mixture was added a solution of crude cyclic imine **9** (1.61 kg, the weight which was determined by HPLC assay method: 1.33 kg, 5.40 mol) in DMA (4.03 kg) dropwise over the course of an hour at 0-12 °C. The additional DMA (0.75 kg) was added for washing. After stirring at 0-5 °C for 4 h, 25% w/w aqueous NH_4Cl solution (18.1 kg) was added to the reaction mixture at 0-15 °C and the resulting mixture was stirred at room temperature for 15 h. The suspension was filtered and the residue was washed with MTBE (4.35 kg). To the combined filtrate was added *n*-heptane (12.0 kg) and the resulting mixture was stirred. After removing the organic phase, the aqueous phase was washed with the solvent mixture of MTBE (4.4 kg) and *n*-heptane (12 kg) four times. Then, the pH of aqueous phase was adjusted to 6.0-6.2 by the addition of 12 M HCl at 20-30 °C. After the addition of NaCl (1.01 kg) to the mixture, pyrrole **15** was extracted with EtOAc (45.6 kg) at 50-53 °C and the resulting organic phase was washed with 25% w/w aqueous NaCl solution (38.7 kg) three times at 50-53 °C. The organic phase was concentrated under reduced pressure and the residue was co-evaporated with 1,2-dimethoxyethane (2.32 kg) twice to afford a crude pyrrole **15** (1.12 kg, the weight which was determined by HPLC assay method: 662 g, 2.85 mol, 53% from **9**).

To a solution of crude pyrrole **15** (1.12 kg, the weight which was determined by HPLC assay method: 659 g, 2.84 mol) in 1,2-dimethoxyethane (7.64 kg) was added *t*-AmOK (0.540 kg, 4.26 mol) at 0-11 °C. After stirring for 40 min, pyridine-3-sulfonylchloride (0.760 kg, 4.26 mol) and 1,2-dimethoxyethane (0.29 kg, for washing the line) were added at 0-10 °C. The reaction mixture was stirred at 0-5 °C for 2.5 h and concentrated under reduced pressure. Next, the residue was diluted with MeOH (15.7 kg) and 12 M HCl (4.67 kg). The resulting mixture was refluxed in an oil bath for 3 h. After cooling to room temperature, the reaction mixture was diluted

with H_2O (22.1 kg) and concentrated to approximately 20 L under reduced pressure. This solution was diluted with H_2O (3.10 kg) and the resulting solution was washed with EtOAc (8.92 kg). After the organic phase was separated, the pH of aqueous phase was adjusted to 4-5 by the addition of 20% w/w aqueous NaOH solution (10.2 kg). Then, EtOAc (9.93 kg) was added. The pH was further adjusted to 10.0-10.5 by the addition of 16% w/w aqueous Na_2CO_3 solution (22.1 kg), Na_2CO_3 (0.73 kg), and 20% w/w aqueous NaOH solution (0.25 kg). After the aqueous phase was separated, the organic phase was washed with 5% w/w aqueous NaCl solution (6.92 kg). The organic phase was concentrated under reduced pressure to afford a crude vonoprazan (**1**) containing EtOAc (1.17 kg, the weight which was determined by HPLC assay method: 741 g, 2.14 mol, 76% over 2 steps from **15**, 23% overall yield from **2**, HPLC purity: 88.8 area%).

4.2. Purification of crude vonoprazan

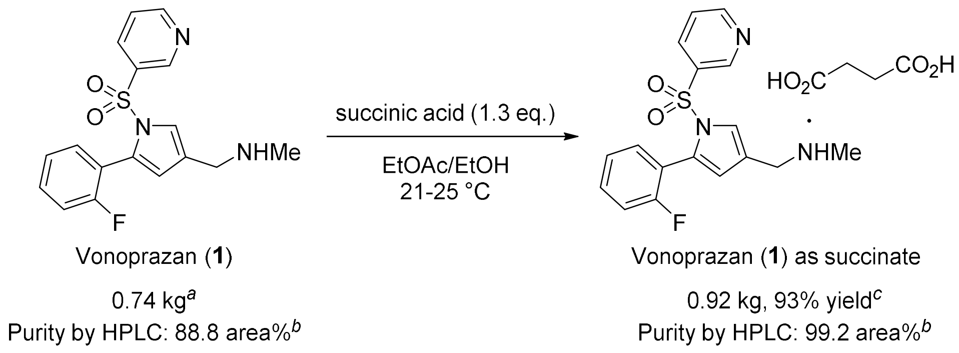

[a]The amount of vonoprazan in the crude product was determined by HPLC assay method which used a calibration curve of the purified vonoprazan. [b]The area% of **1** relative to the total area of all peaks in the crude or purified product was determined by HPLC. [c]Isolated yield.

Scheme S8. Purification of crude vonoprazan (1)

In order to purify the crude vonoprazan (**1**) obtained by large-scale synthesis, the purification methods were investigated. As shown in Scheme S8, it turned out that the crystallization process as succinate was highly effective. The following procedure afforded the purified vonoprazan with the HPLC purity of 99.2 area% in 93% yield.

To a solution of crude vonoprazan (**1**) containing EtOAc (1.17 kg, the weight which was determined by HPLC assay method: 741 g, 2.14 mol, HPLC purity: 88.8 area%) in EtOAc (2.59 kg) was added the solution of succinic acid (99.5 g, 0.842 mol) in EtOH (1.06 kg) at 21-23 °C. The resulting suspension was stirred at 21-23 °C for 21 h, and then the additional solution of succinic acid (230 g, 1.95 mol) in EtOH (2.49 kg) was added dropwise over the course of 46 minutes at 22-25 °C. After washing the drip line by using EtOH (0.595 kg), the resulting suspension was stirred at 21-23 °C for 23 h. The wet product was collected by filtration and washed with EtOH (1.17 kg) 5 times. After drying the wet product under reduced pressure at 40 °C for 19 h, vonoprazan succinate was obtained as slightly brown solid (923 g, 1.99 mol, 93%, HPLC purity: 99.2 area%).

4.3. HPLC chromatograms of intermediates, crude vonoprazan, and purified vonoprazan

HPLC chromatograms of intermediates, crude vonoprazan, and purified vonoprazan are provided.

HPLC chromatogram of compound 11 (crude)

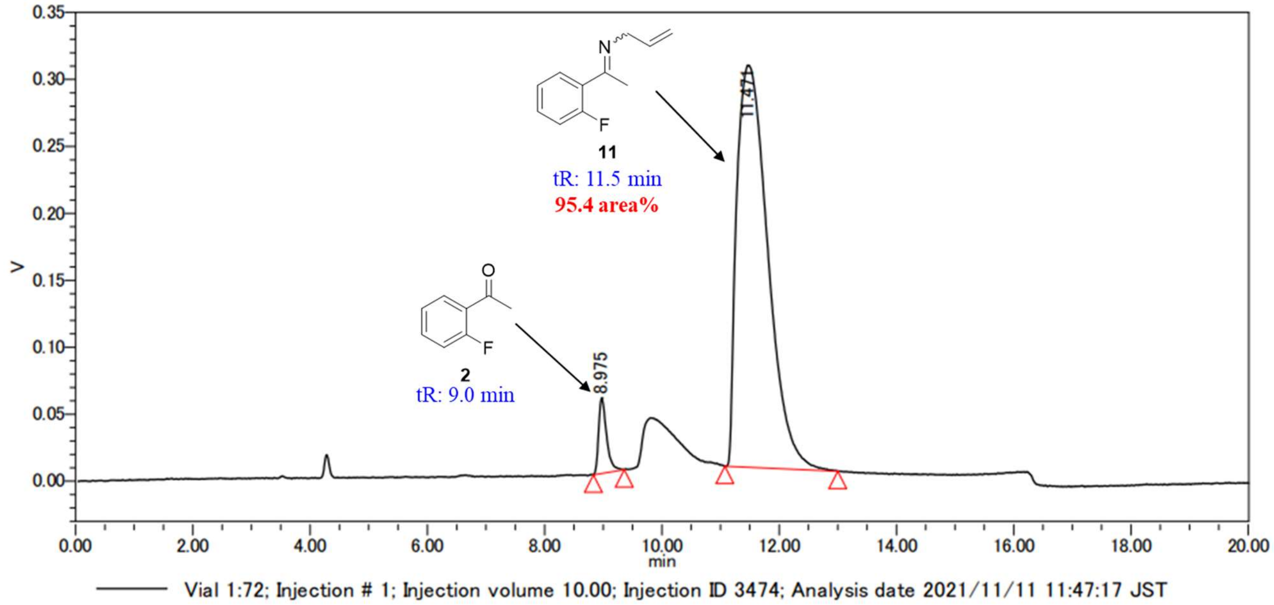

	tR	Area	Area%
1	8.975	533768	4.61
2	11.471	11051997	95.39

The following HPLC conditions were used. HPLC (IB-3, *n*-heptane/diethylamine = 100/0.05, flow rate = 1.0 mL/min, l = 210 nm), tR = 11.5 min (compound **11**), 9.0 min (compound **2**). The peaks of solvents and blank were not integrated.

Other conditions and information are as follows.
Column: CHIRALPAK IB-3 4.6 x 250 mm (3 μm) manufactured by Daicel Corporation
Temperature of column oven: 30 °C
Analysis time: 20 min

HPLC chromatogram of compound 12 (crude)

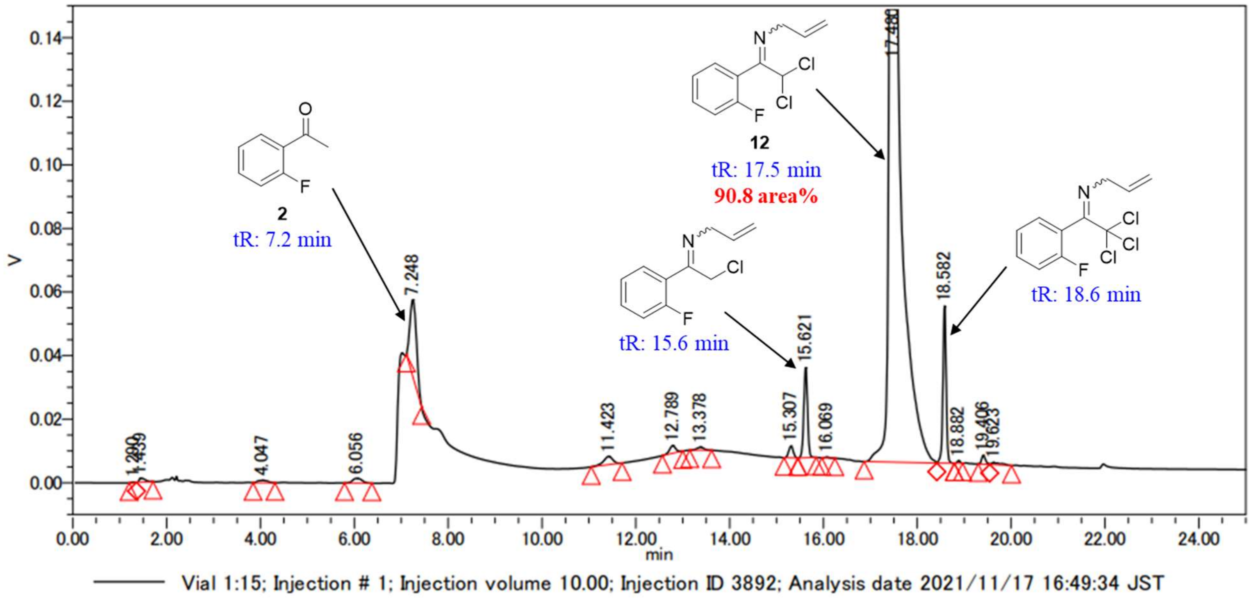

	tR	Area	Area%
1	1.290	818	0.01
2	1.439	11292	0.13
3	4.047	10880	0.12
4	6.056	20460	0.24
5	7.248	238394	2.74
6	11.423	35451	0.41
7	12.789	21764	0.25
8	13.378	10275	0.12
9	15.307	25296	0.29
10	15.621	157866	1.81
11	16.069	1831	0.02
12	17.480	7904332	90.81
13	18.582	236266	2.71
14	18.882	3485	0.04
15	19.406	15912	0.18
16	19.623	9786	0.11

The following HPLC conditions were used. HPLC (CN-3, gradient method by using A: 10 mM sodium phosphate buffer (pH 7.1) and B: MeCN, flow rate = 1.0 mL/min, l = 225 nm), tR = 17.5 min (compound **12**), 7.2 min (compound **2**). The peaks of solvents and blank were not integrated.

Other conditions and information are as follows.

Gradient method: The gradient of concentration was controlled by changing the mixing ratios of mobile phase A and B as follows.

Time (min) after injection	A (%)	B (%)
0	90	10
5	90	10
20	20	80
25	20	80

Column: Inertsil CN-3 4.6 x 150 mm (3 μm) manufactured by GL Sciences Inc.

Temperature of column oven: 30 °C

Analysis time: 25 min

HPLC chromatogram of compound 9 (crude)

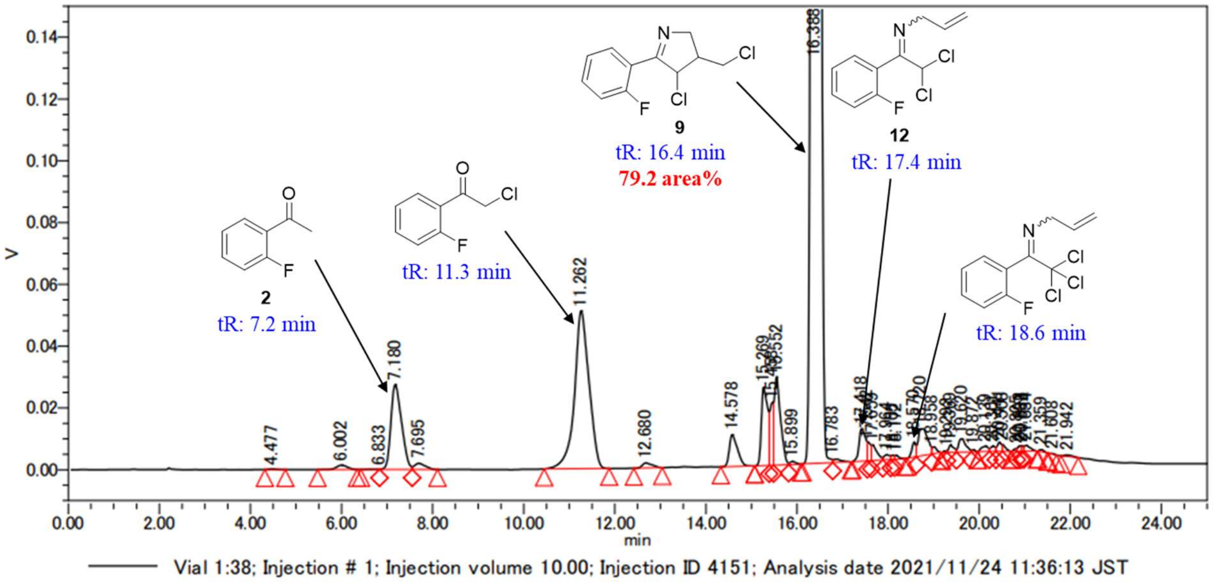

	tR	Area	Area%
1	4.477	2755	0.02
2	6.002	23319	0.16
3	6.833	1417	0.01
4	7.180	446510	3.10
5	7.695	32845	0.23
6	11.262	1131904	7.87
7	12.680	24389	0.17
8	14.578	128747	0.89
9	15.269	244084	1.70
10	15.458	105605	0.73
11	15.552	274638	1.91
12	15.899	8732	0.06
13	16.388	11400332	79.23
14	16.783	13275	0.09
15	17.418	104381	0.73
16	17.542	30533	0.21
17	17.659	41544	0.29
18	17.964	13593	0.09
19	18.105	6260	0.04
20	18.172	7570	0.05
21	18.570	28457	0.20
22	18.720	125628	0.87
23	18.958	14946	0.10

	tR	Area	Area%
24	19.292	2271	0.02
25	19.369	18291	0.13
26	19.620	46720	0.32
27	19.872	3231	0.02
28	20.139	17920	0.12
29	20.301	12394	0.09
30	20.441	19397	0.13
31	20.508	10588	0.07
32	20.825	3684	0.03
33	20.893	8923	0.06
34	20.938	5759	0.04
35	21.034	16662	0.12
36	21.359	4219	0.03
37	21.608	995	0.01
38	21.942	6060	0.04

The HPLC conditions were same as those of compound **12**. tR = 16.4 min (compound **9**), 17.4 min (compound **12**). The peaks of solvents and blank were not integrated.

HPLC chromatogram of compound 15 (crude)

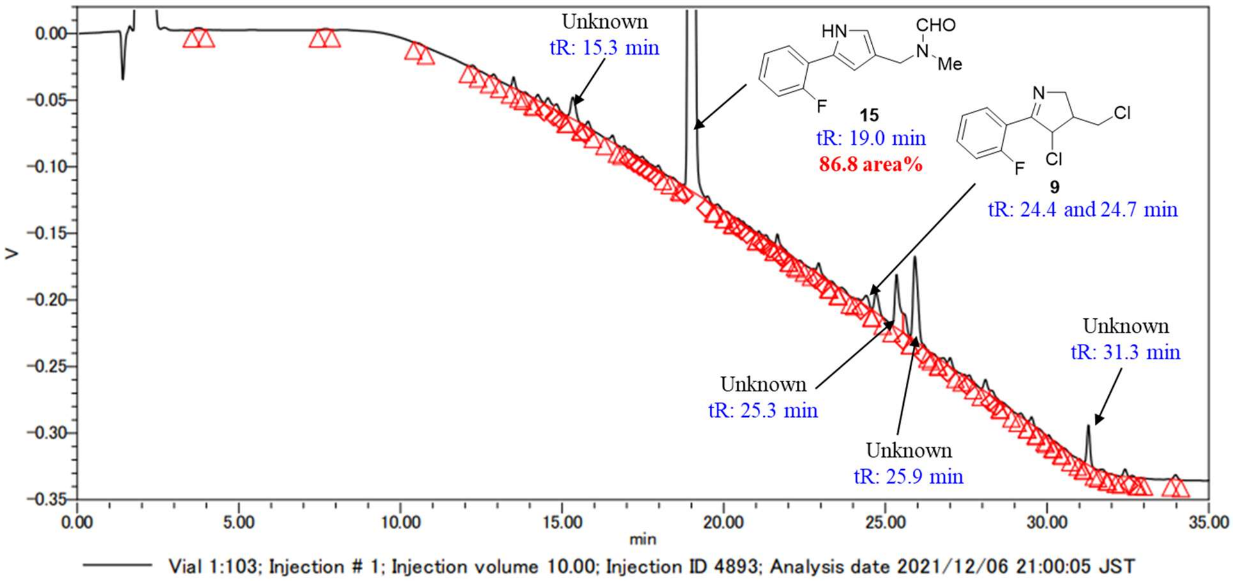

	tR	Area	Area%
1	3.747	23038	0.10
2	7.685	12734	0.05
3	10.592	9921	0.04
4	12.250	17736	0.08
5	12.883	22812	0.10
6	13.498	49078	0.21
7	13.861	10619	0.05
8	14.248	40307	0.17
9	14.558	66611	0.28
10	14.828	41445	0.18
11	14.942	5471	0.02
12	15.334	173112	0.74
13	15.708	1857	0.01
14	15.801	33293	0.14
15	16.558	45802	0.20
16	16.922	20412	0.09
17	17.100	17631	0.08
18	17.308	7712	0.03
19	17.479	21839	0.09
20	17.600	9577	0.04
21	17.800	8602	0.04
22	17.976	24428	0.10
23	18.450	11025	0.05

	tR	Area	Area%
24	18.792	3733	0.02
25	18.975	20321232	86.83
26	19.500	28172	0.12
27	19.802	20198	0.09
28	20.133	9250	0.04
29	20.392	2596	0.01
30	20.458	10723	0.05
31	20.608	12611	0.05
32	20.808	10950	0.05
33	21.088	14414	0.06
34	21.242	4940	0.02
35	21.309	20300	0.09
36	21.475	2757	0.01
37	21.660	54820	0.23
38	21.750	12179	0.05
39	21.892	10474	0.04
40	22.092	2283	0.01
41	22.375	9312	0.04
42	22.842	8019	0.03
43	22.934	62770	0.27
44	23.067	6979	0.03
45	23.367	11919	0.05
46	23.675	27296	0.12

	tR	Area	Area%
47	24.192	11826	0.05
48	24.406	85459	0.37
49	24.711	141511	0.60
50	25.347	419247	1.79
51	25.582	104933	0.45
52	25.914	643271	2.75
53	26.183	10883	0.05
54	26.525	6180	0.03
55	26.817	30623	0.13
56	27.002	50578	0.22
57	27.467	7434	0.03
58	27.593	21787	0.09
59	28.096	77701	0.33
60	28.292	37014	0.16
61	28.417	4304	0.02
62	28.675	12434	0.05
63	29.200	21090	0.09
64	29.521	36199	0.15
65	29.792	8731	0.04
66	30.067	11993	0.05
67	30.317	5966	0.03
68	30.575	8341	0.04
69	31.025	2988	0.01

	tR	Area	Area%
70	31.280	197369	0.84
71	31.718	16310	0.07
72	31.975	3411	0.01
73	32.413	39617	0.17
74	32.632	10444	0.04
75	32.893	2367	0.01
76	33.976	29173	0.12

The following HPLC conditions were used. HPLC (C18, gradient method by using A: 10 mM ammonium formate aqueous solution and B: MeCN, flow rate = 0.77 mL/min, l = 210 nm), tR = 19.0 min (compound **15**), 24.4 and 24.7 min (compound **9**). The peaks of solvents and blank were not integrated. In order to make the peaks visible, the retention times of integrated peaks were not indicated on the chromatogram.

Other conditions and information are as follows.

Gradient method: The gradient of concentration was controlled by changing the mixing ratios of mobile phase A and B as follows.

Time (min) after injection	A (%)	B (%)
0	90	10
4	90	10
27	20	80
31	20	80

Column: ZORBAX Eclipse Plus C18 4.6 x 100 mm (3.5 µm) manufactured by Agilent Technologies, Inc.

Temperature of column oven: 30 °C

Analysis time: 35 min

HPLC chromatogram of crude vonoprazan (1)

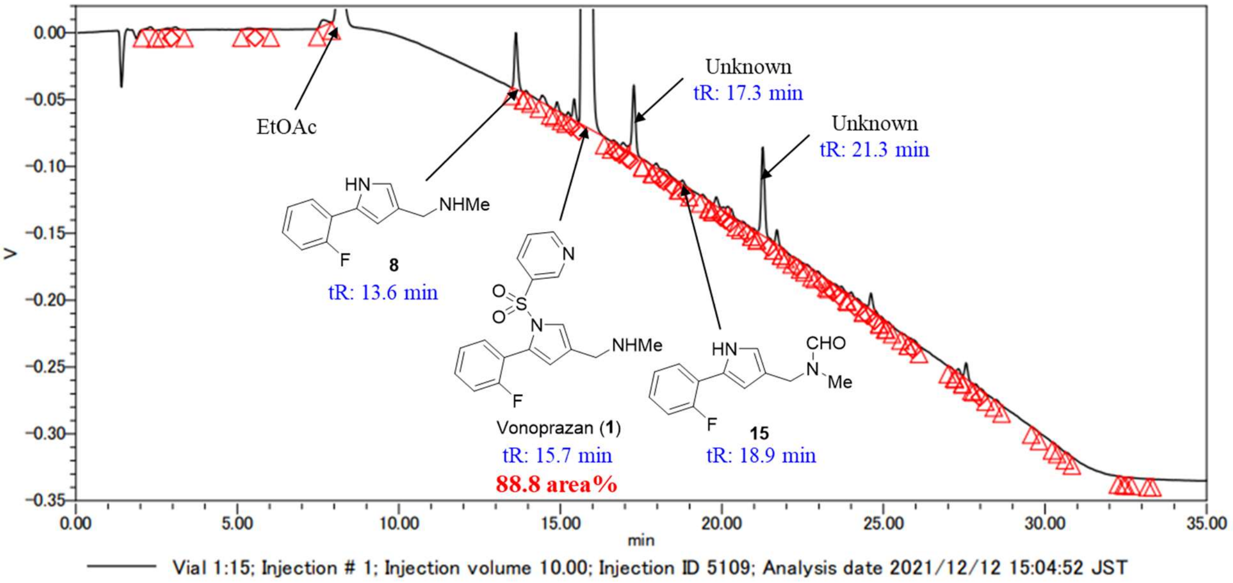

	tR	Area	Area%
1	2.314	26245	0.13
2	2.844	14473	0.07
3	3.084	22251	0.11
4	5.363	14952	0.07
5	5.832	12391	0.06
6	7.633	65345	0.32
7	13.624	297863	1.44
8	13.936	12180	0.06
9	14.450	53092	0.26
10	14.896	37764	0.18
11	15.244	22322	0.11
12	15.425	98897	0.48
13	15.729	18382068	88.78
14	16.658	11540	0.06
15	16.717	6611	0.03
16	16.942	41336	0.20
17	17.150	18778	0.09
18	17.274	370724	1.79
19	17.658	6227	0.03
20	17.965	28451	0.14
21	18.208	15386	0.07
22	18.275	32065	0.15
23	18.475	4487	0.02

	tR	Area	Area%
24	18.777	23124	0.11
25	18.900	1566	0.01
26	19.417	14196	0.07
27	19.650	700	0.00
28	19.826	49107	0.24
29	20.117	20234	0.10
30	20.182	37661	0.18
31	20.268	30001	0.14
32	20.675	10102	0.05
33	20.808	3741	0.02
34	21.000	5726	0.03
35	21.267	481276	2.32
36	21.483	6785	0.03
37	21.702	62719	0.30
38	21.900	2430	0.01
39	22.250	2659	0.01
40	22.358	2328	0.01
41	22.667	7874	0.04
42	22.992	10730	0.05
43	23.125	3025	0.01
44	23.375	1547	0.01
45	23.542	6883	0.03
46	23.717	16828	0.08

	tR	Area	Area%
47	24.001	18569	0.09
48	24.233	10775	0.05
49	24.450	4227	0.02
50	24.612	84030	0.41
51	24.725	10739	0.05
52	24.958	5626	0.03
53	25.175	6014	0.03
54	25.658	4064	0.02
55	25.917	6668	0.03
56	25.975	11427	0.06
57	27.092	3732	0.02
58	27.319	21807	0.11
59	27.558	77247	0.37
60	27.875	10927	0.05
61	28.058	7301	0.04
62	28.525	9340	0.05
63	29.700	3818	0.02
64	30.292	3285	0.02
65	30.725	5492	0.03
66	32.333	872	0.00
67	32.569	1184	0.01
68	33.267	485	0.00

The HPLC conditions were same as those of compound **15**. tR = 15.7 min (vonoprazan **1**). The peaks of solvents and blank were not integrated. In order to make the peaks visible, the retention times of integrated peaks were not indicated on the chromatogram.

HPLC chromatogram of purified vonoprazan

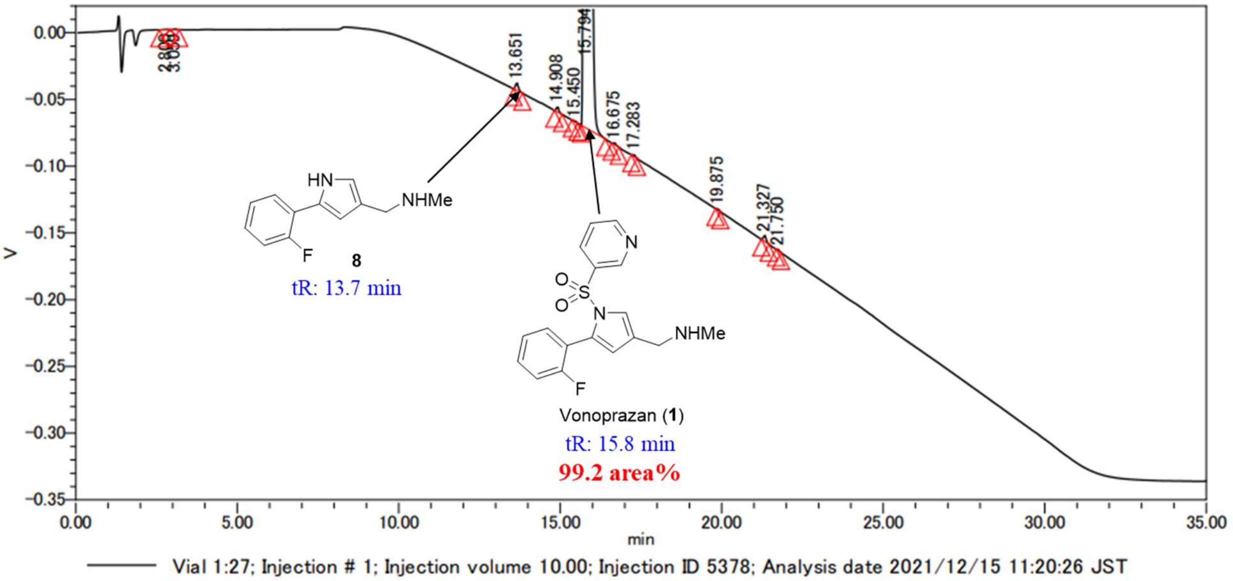

	tR	Area	Area%
1	2.800	3419	0.03
2	3.059	3331	0.03
3	13.651	34103	0.27
4	14.908	19632	0.16
5	15.450	2095	0.02
6	15.794	12540510	99.15
7	16.675	6825	0.05
8	17.283	6268	0.05
9	19.875	2485	0.02
10	21.327	25746	0.20
11	21.750	3064	0.02

The HPLC conditions were same as those of compound **15**. tR = 15.8 min (vonoprazan **1**). The peaks of succinic acid and blank were not integrated.

5. NMR Spectra

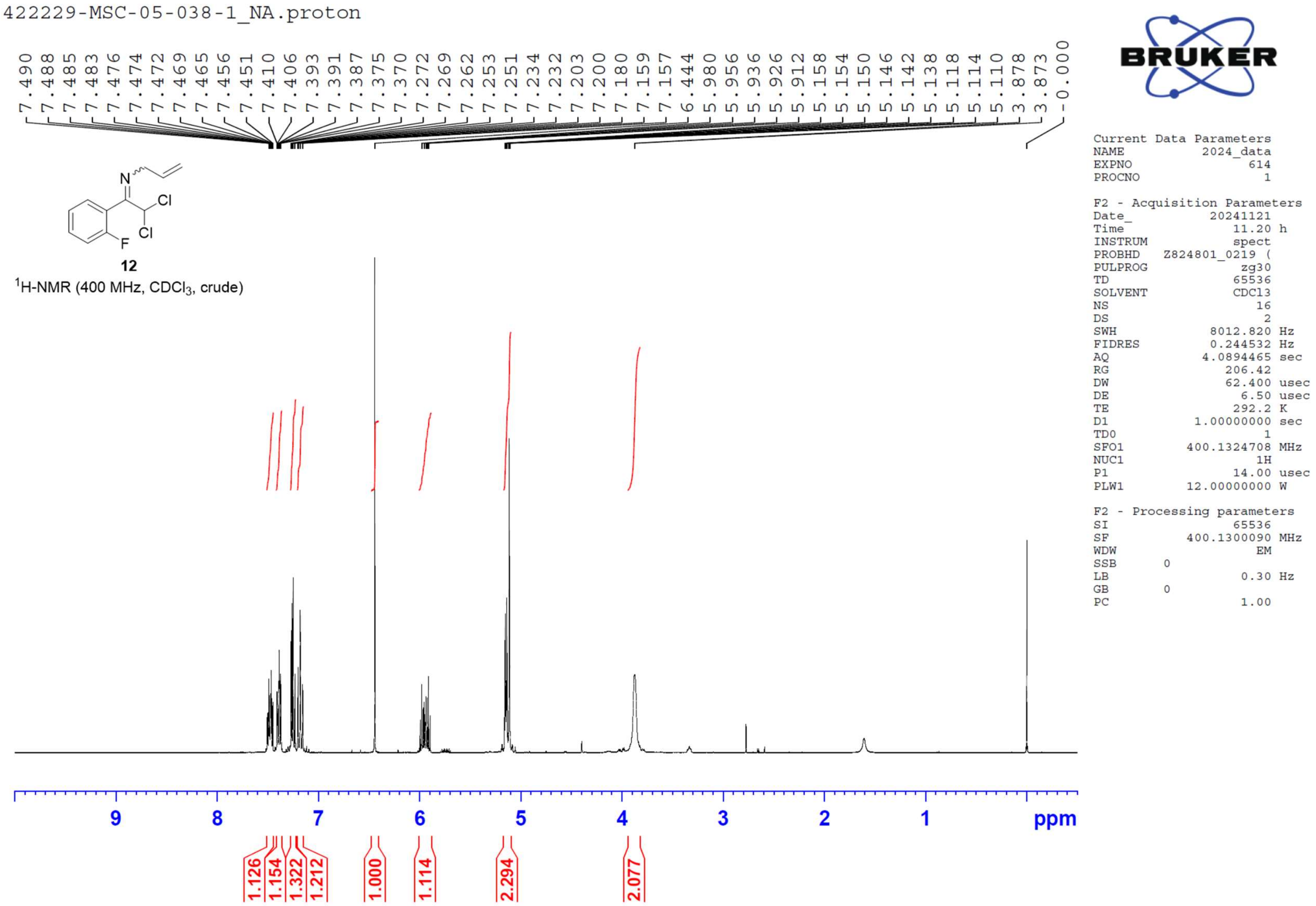

422229-MSC-05-038-1_NA.proton
7.490 7.488 7.485 7.483 7.476 7.474 7.472 7.469 7.465 7.456 7.451 7.410 7.406 7.393 7.391 7.387 7.375 7.370 7.272 7.269 7.262 7.253 7.251 7.234 7.232 7.203 7.200 7.180 7.159 7.157 6.444 5.980 5.956 5.936 5.926 5.912 5.158 5.154 5.150 5.146 5.142 5.138 5.118 5.114 5.110 3.878 3.873 -0.000
12
^{1}H-NMR (400 MHz, CDCl$_3$, crude)
1.126 1.154 1.322 1.212 1.000 1.114 2.294 2.077
9 8 7 6 5 4 3 2 1 ppm
BRUKER
Current Data Parameters
NAME 2024_data
EXPNO 614
PROCNO 1
F2 - Acquisition Parameters
Date_ 20241121
Time 11.20 h
INSTRUM spect
PROBHD Z824801_0219 (
PULPROG zg30
TD 65536
SOLVENT CDCl3
NS 16
DS 2
SWH 8012.820 Hz
FIDRES 0.244532 Hz
AQ 4.0894465 sec
RG 206.42
DW 62.400 usec
DE 6.50 usec
TE 292.2 K
D1 1.00000000 sec
TD0 1
SFO1 400.1324708 MHz
NUC1 1H
P1 14.00 usec
PLW1 12.00000000 W
F2 - Processing parameters
SI 65536
SF 400.1300090 MHz
WDW EM
SSB 0
LB 0.30 Hz
GB 0
PC 1.00

422229-MSC-05-038-1_NA.carbon

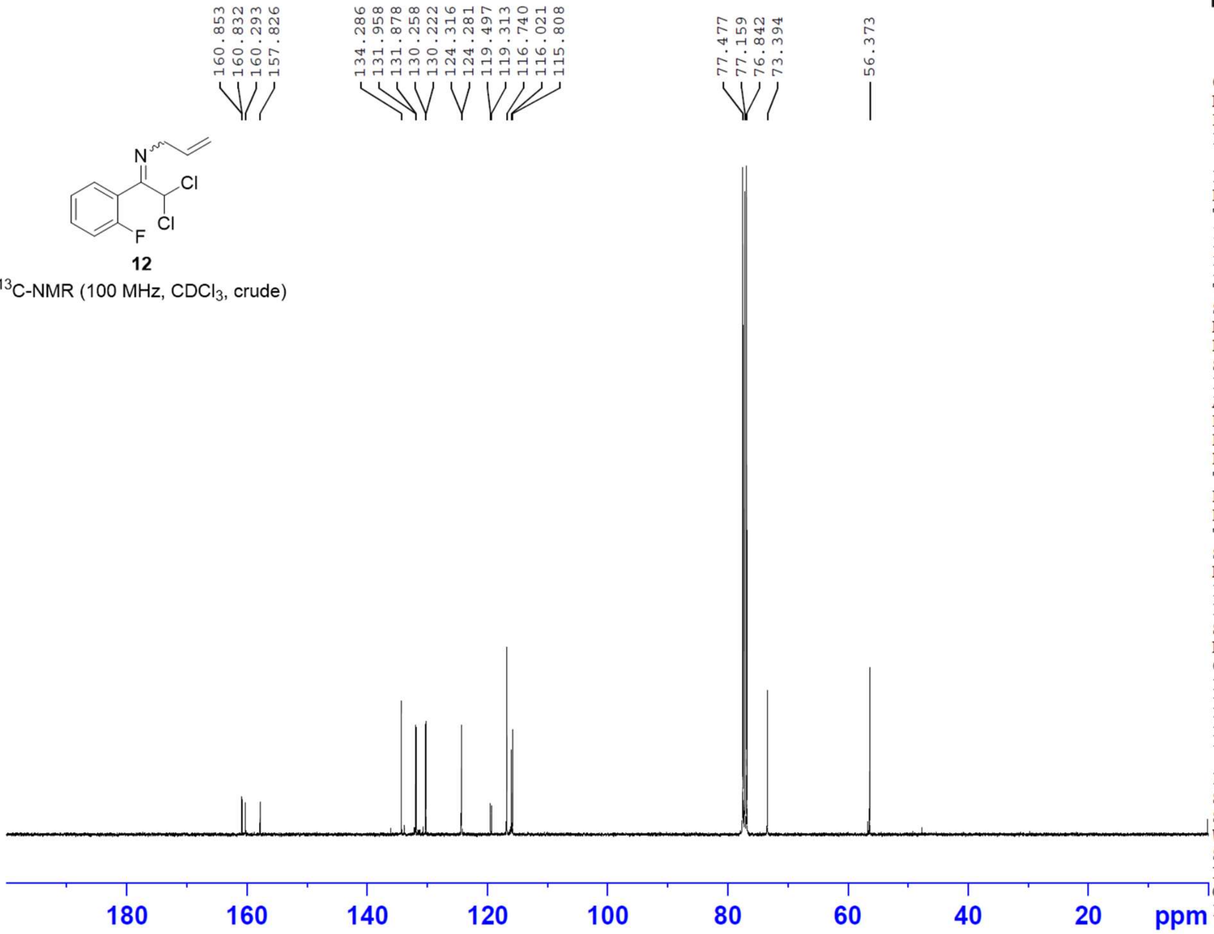

12

^{13}C-NMR (100 MHz, $CDCl_3$, crude)

Current Data Parameters
NAME 2024_data
EXPNO 615
PROCNO 1

F2 - Acquisition Parameters
Date_ 20241121
Time 15.15 h
INSTRUM spect
PROBHD Z824801_0219 (
PULPROG zgpg30
TD 65536
SOLVENT CDCl3
NS 4096
DS 4
SWH 24038.461 Hz
FIDRES 0.733596 Hz
AQ 1.3631488 sec
RG 206.42
DW 20.800 usec
DE 6.50 usec
TE 293.2 K
D1 2.00000000 sec
D11 0.03000000 sec
TD0 1
SFO1 100.6228298 MHz
NUC1 13C
P1 10.00 usec
PLW1 50.00000000 W
SFO2 400.1316005 MHz
NUC2 1H
CPDPRG[2 waltz16
PCPD2 90.00 usec
PLW2 12.00000000 W
PLW12 0.29036999 W
PLW13 0.14605001 W

F2 - Processing parameters
SI 32768
SF 100.6127564 MHz
WDW EM
SSB 0
LB 1.00 Hz
GB 0
PC 1.40

TR-109-VPZ_N A.proton_Ojima

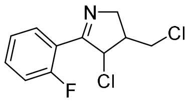

9

^{1}H-NMR (400 MHz, $CDCl_3$, a mixture of diastereomers)

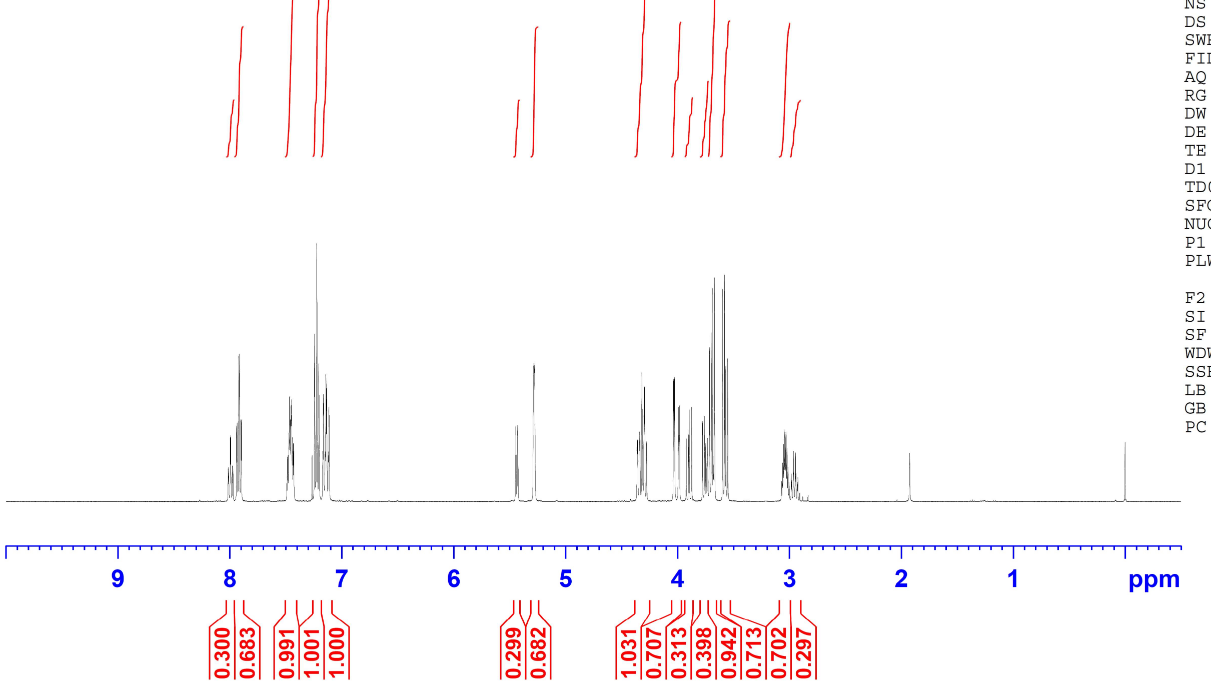

BRUKER

```
Current Data Parameters
NAME            2022_data
EXPNO                 435
PROCNO                  1

F2 - Acquisition Parameters
Date_            20221208
Time                11.31 h
INSTRUM             spect
PROBHD   Z108618_0899 (
PULPROG              zg30
TD                  65536
SOLVENT             CDCl3
NS                      8
DS                      2
SWH              8012.820 Hz
FIDRES           0.244532 Hz
AQ              4.0894465 sec
RG                  91.71
DW                 62.400 usec
DE                   6.50 usec
TE                  293.7 K
D1             1.00000000 sec
TD0                     1
SFO1          400.1324708 MHz
NUC1                   1H
P1                  14.00 usec
PLW1          15.91600037 W

F2 - Processing parameters
SI                  65536
SF            400.1300076 MHz
WDW                    EM
SSB      0
LB                   0.30 Hz
GB       0
PC                   1.00
```

TR-109-VPZ_N A.carbon13_Ojima

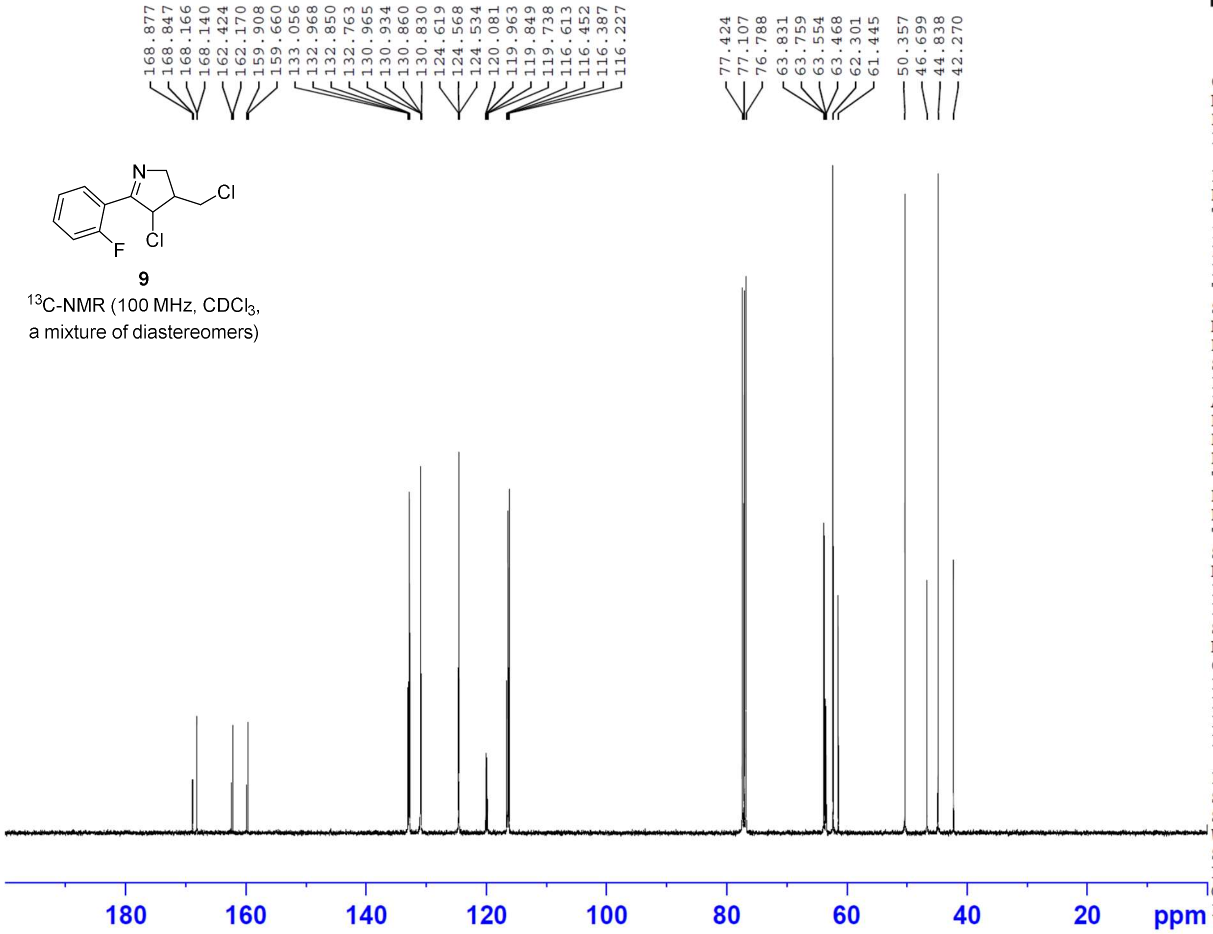

9

^{13}C-NMR (100 MHz, $CDCl_3$, a mixture of diastereomers)

Current Data Parameters
NAME 2022_data
EXPNO 436
PROCNO 1

F2 - Acquisition Parameters
Date_ 20221208
Time 12.30 h
INSTRUM spect
PROBHD Z108618_0899 (
PULPROG zgpg30
TD 65536
SOLVENT CDCl3
NS 1024
DS 4
SWH 24038.461 Hz
FIDRES 0.733596 Hz
AQ 1.3631488 sec
RG 206.42
DW 20.800 usec
DE 6.50 usec
TE 295.0 K
D1 2.00000000 sec
D11 0.03000000 sec
TD0 1
SFO1 100.6228298 MHz
NUC1 13C
P1 10.00 usec
PLW1 70.63899994 W
SFO2 400.1316005 MHz
NUC2 1H
CPDPRG[2 waltz16
PCPD2 90.00 usec
PLW2 15.91600037 W
PLW12 0.38512000 W
PLW13 0.19371000 W

F2 - Processing parameters
SI 32768
SF 100.6127685 MHz
WDW EM
SSB 0
LB 1.00 Hz
GB 0
PC 1.40

TR-110-VPZ_N A.proton_Ojima

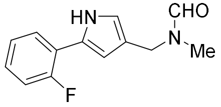

15

^{1}H-NMR (400 MHz, $CDCl_3$, a mixture of rotamers)

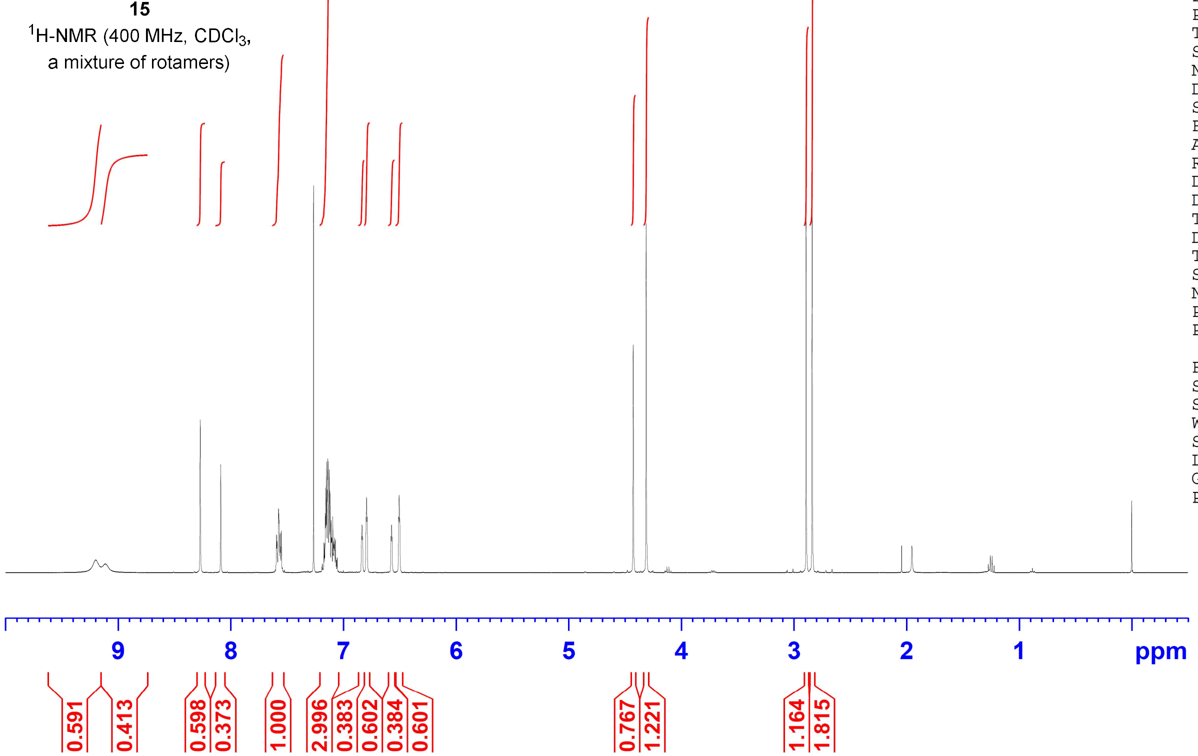

Current Data Parameters
NAME 2022_data
EXPNO 437
PROCNO 1

F2 - Acquisition Parameters
Date_ 20221208
Time 13.14 h
INSTRUM spect
PROBHD Z108618_0899 (
PULPROG zg30
TD 65536
SOLVENT CDCl3
NS 8
DS 2
SWH 8012.820 Hz
FIDRES 0.244532 Hz
AQ 4.0894465 sec
RG 149.18
DW 62.400 usec
DE 6.50 usec
TE 293.9 K
D1 1.00000000 sec
TD0 1
SFO1 400.1324708 MHz
NUC1 1H
P1 14.00 usec
PLW1 15.91600037 W

F2 - Processing parameters
SI 65536
SF 400.1300075 MHz
WDW EM
SSB 0
LB 0.30 Hz
GB 0
PC 1.00

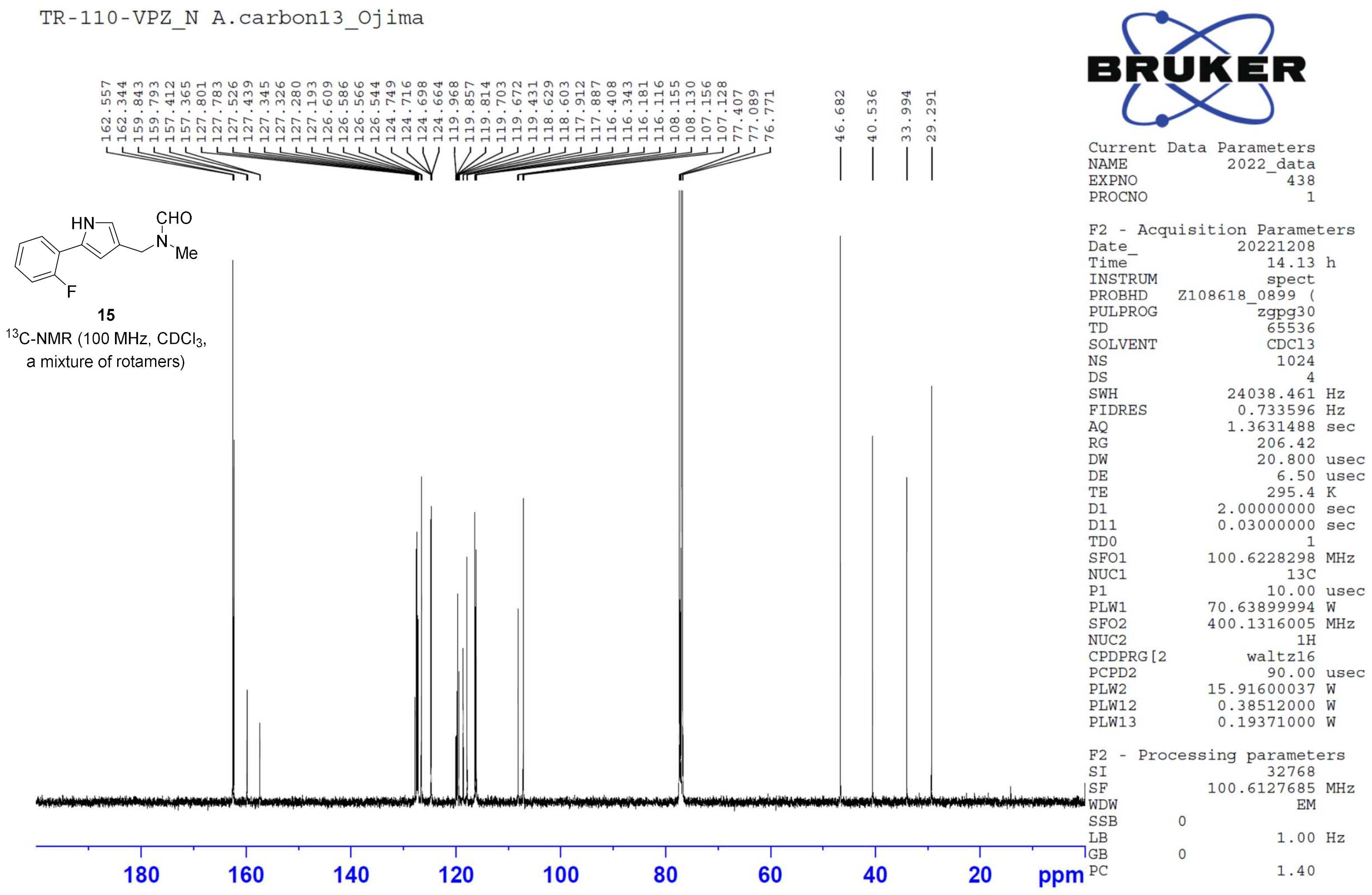

TR-110-VPZ_N A.carbon13_Ojima
162.557
162.344
159.843
159.793
157.412
157.365
127.801
127.783
127.526
127.439
127.345
127.326
127.280
127.193
126.609
126.586
126.566
126.544
124.749
124.716
124.698
124.664
119.968
119.857
119.814
119.703
119.672
119.431
118.629
118.603
117.912
117.887
116.408
116.343
116.181
116.116
108.155
108.130
107.156
107.128
77.407
77.089
76.771
46.682
40.536
33.994
29.291
HN
CHO
N
Me
F
15
^{13}C-NMR (100 MHz, CDCl$_3$,
a mixture of rotamers)
180
160
140
120
100
80
60
40
20
ppm
BRUKER
Current Data Parameters
NAME 2022_data
EXPNO 438
PROCNO 1
F2 - Acquisition Parameters
Date_ 20221208
Time 14.13 h
INSTRUM spect
PROBHD Z108618_0899 (
PULPROG zgpg30
TD 65536
SOLVENT CDCl3
NS 1024
DS 4
SWH 24038.461 Hz
FIDRES 0.733596 Hz
AQ 1.3631488 sec
RG 206.42
DW 20.800 usec
DE 6.50 usec
TE 295.4 K
D1 2.00000000 sec
D11 0.03000000 sec
TD0 1
SFO1 100.6228298 MHz
NUC1 13C
P1 10.00 usec
PLW1 70.63899994 W
SFO2 400.1316005 MHz
NUC2 1H
CPDPRG[2 waltz16
PCPD2 90.00 usec
PLW2 15.91600037 W
PLW12 0.38512000 W
PLW13 0.19371000 W
F2 - Processing parameters
SI 32768
SF 100.6127685 MHz
WDW EM
SSB 0
LB 1.00 Hz
GB 0
PC 1.40

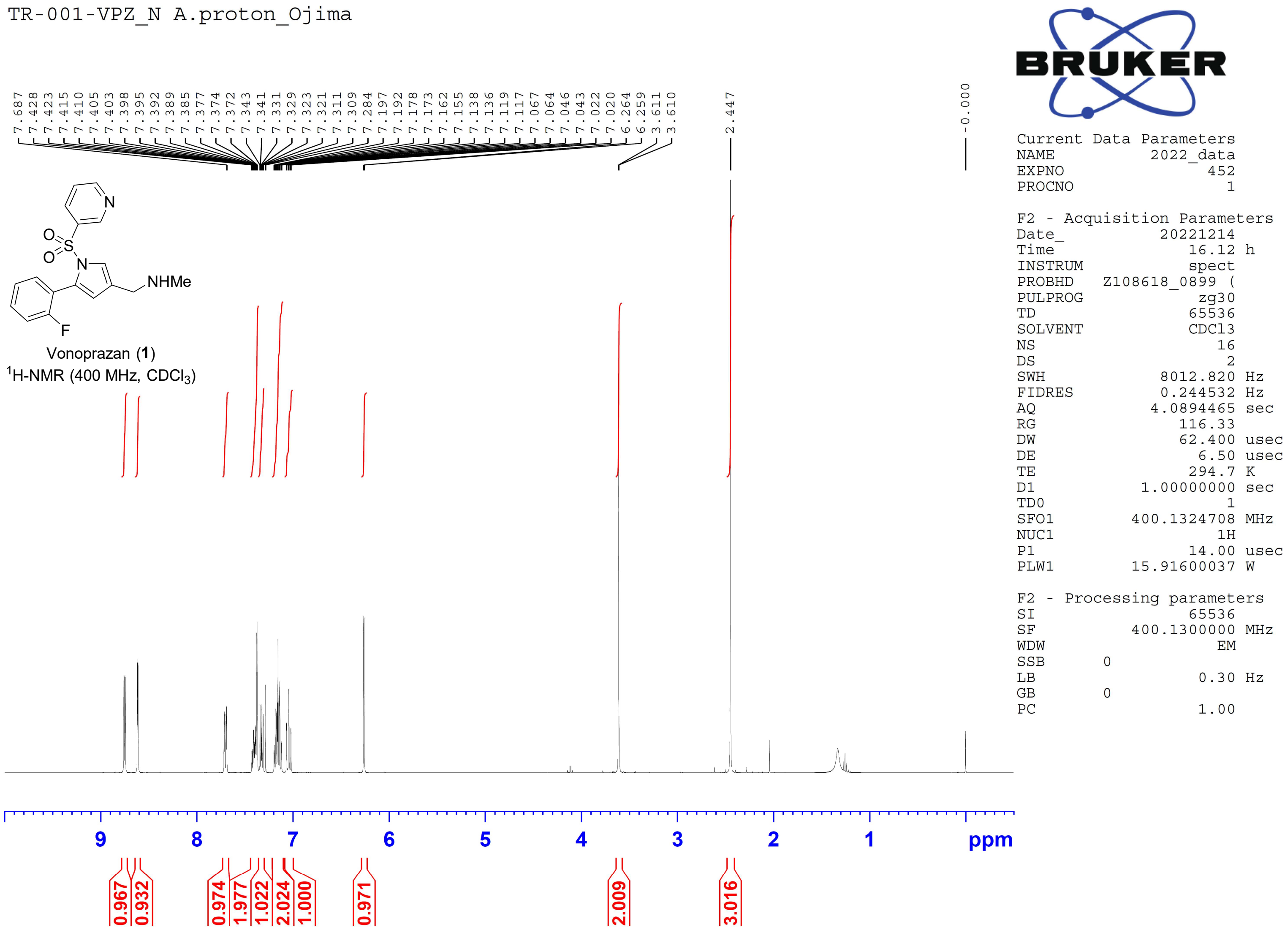
TR-001-VPZ_N A.proton_Ojima
7.687
7.428
7.423
7.415
7.410
7.405
7.403
7.398
7.395
7.392
7.389
7.385
7.377
7.374
7.372
7.343
7.341
7.331
7.329
7.323
7.321
7.311
7.309
7.284
7.197
7.192
7.178
7.173
7.162
7.155
7.138
7.136
7.119
7.117
7.067
7.064
7.046
7.043
7.022
7.020
6.264
6.259
3.611
3.610
2.447
-0.000
N
O
S
O
N
NHMe
F
Vonoprazan (1)
1H-NMR (400 MHz, CDCl3)
BRUKER
Current Data Parameters
NAME 2022_data
EXPNO 452
PROCNO 1
F2 - Acquisition Parameters
Date_ 20221214
Time 16.12 h
INSTRUM spect
PROBHD Z108618_0899 (
PULPROG zg30
TD 65536
SOLVENT CDCl3
NS 16
DS 2
SWH 8012.820 Hz
FIDRES 0.244532 Hz
AQ 4.0894465 sec
RG 116.33
DW 62.400 usec
DE 6.50 usec
TE 294.7 K
D1 1.00000000 sec
TD0 1
SFO1 400.1324708 MHz
NUC1 1H
P1 14.00 usec
PLW1 15.91600037 W
F2 - Processing parameters
SI 65536
SF 400.1300000 MHz
WDW EM
SSB 0
LB 0.30 Hz
GB 0
PC 1.00
9
8
7
6
5
4
3
2
1
ppm
0.967
0.932
0.974
1.977
1.022
2.024
1.000
0.971
2.009
3.016

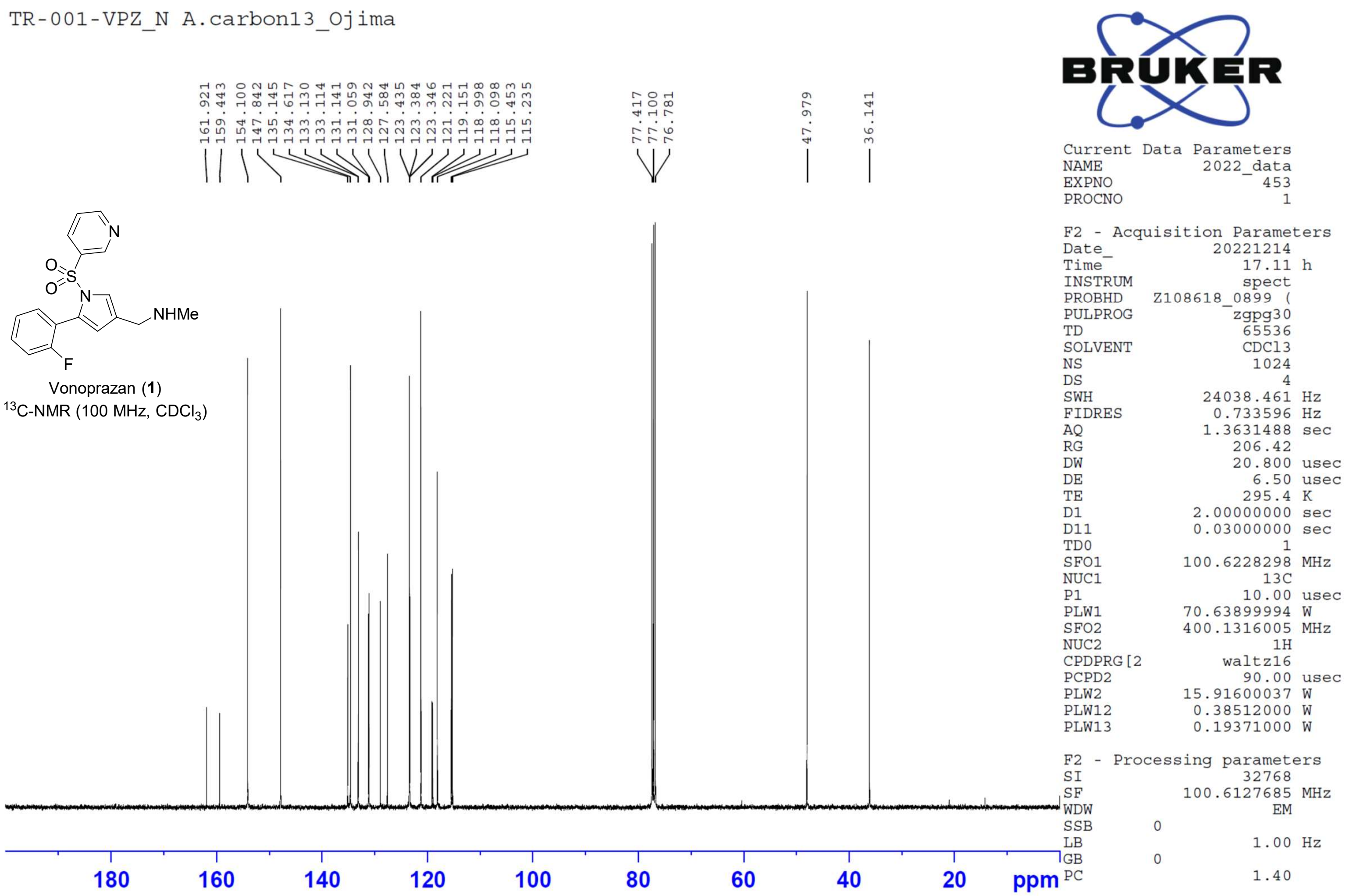

TR-001-VPZ_N A.carbon13_Ojima
161.921
159.443
154.100
147.842
135.145
134.617
133.130
133.114
131.141
131.059
128.942
127.584
123.435
123.384
123.346
121.221
119.151
118.998
118.098
115.453
115.235
77.417
77.100
76.781
47.979
36.141
N
O
S
O
N
NHMe
F
Vonoprazan (1)
13C-NMR (100 MHz, CDCl3)
BRUKER
Current Data Parameters
NAME 2022_data
EXPNO 453
PROCNO 1
F2 - Acquisition Parameters
Date_ 20221214
Time 17.11 h
INSTRUM spect
PROBHD Z108618_0899 (
PULPROG zgpg30
TD 65536
SOLVENT CDCl3
NS 1024
DS 4
SWH 24038.461 Hz
FIDRES 0.733596 Hz
AQ 1.3631488 sec
RG 206.42
DW 20.800 usec
DE 6.50 usec
TE 295.4 K
D1 2.00000000 sec
D11 0.03000000 sec
TD0 1
SFO1 100.6228298 MHz
NUC1 13C
P1 10.00 usec
PLW1 70.63899994 W
SFO2 400.1316005 MHz
NUC2 1H
CPDPRG[2 waltz16
PCPD2 90.00 usec
PLW2 15.91600037 W
PLW12 0.38512000 W
PLW13 0.19371000 W
F2 - Processing parameters
SI 32768
SF 100.6127685 MHz
WDW EM
SSB 0
LB 1.00 Hz
GB 0
PC 1.40
180
160
140
120
100
80
60
40
20
ppm

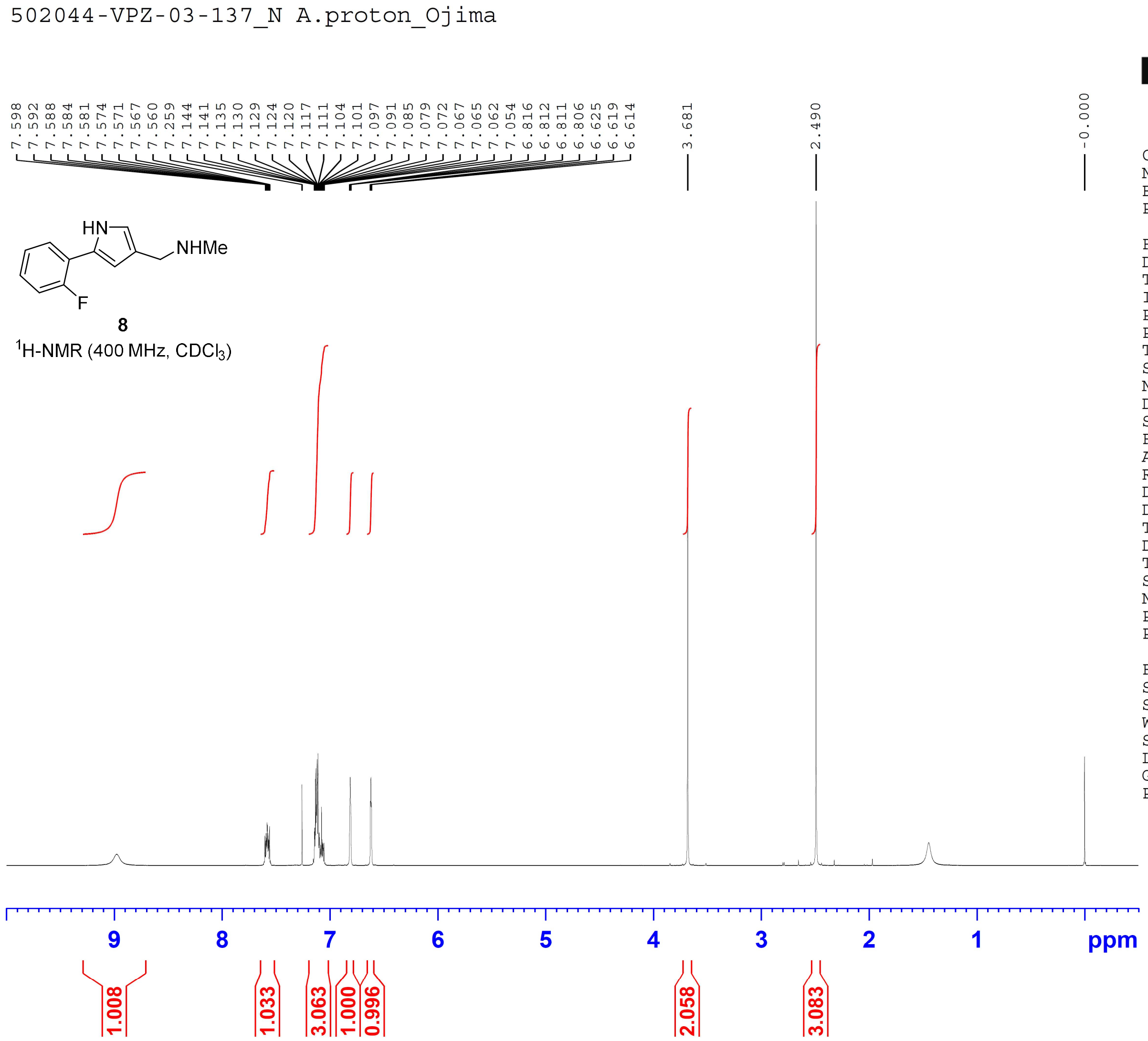

BRUKER

Current Data Parameters
NAME 2022_data
EXPNO 454
PROCNO 1

F2 - Acquisition Parameters
Date_ 20221214
Time 17.15 h
INSTRUM spect
PROBHD Z108618_0899 (
PULPROG zg30
TD 65536
SOLVENT CDCl3
NS 16
DS 2
SWH 8012.820 Hz
FIDRES 0.244532 Hz
AQ 4.0894465 sec
RG 206.42
DW 62.400 usec
DE 6.50 usec
TE 294.4 K
D1 1.00000000 sec
TD0 1
SFO1 400.1324708 MHz
NUC1 1H
P1 14.00 usec
PLW1 15.91600037 W

F2 - Processing parameters
SI 65536
SF 400.1300101 MHz
WDW EM
SSB 0
LB 0.30 Hz
GB 0
PC 1.00

502044-VPZ-03-137_N A.carbon13_Ojima

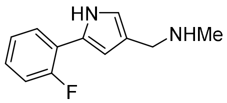

8

^{13}C-NMR (100 MHz, $CDCl_3$)

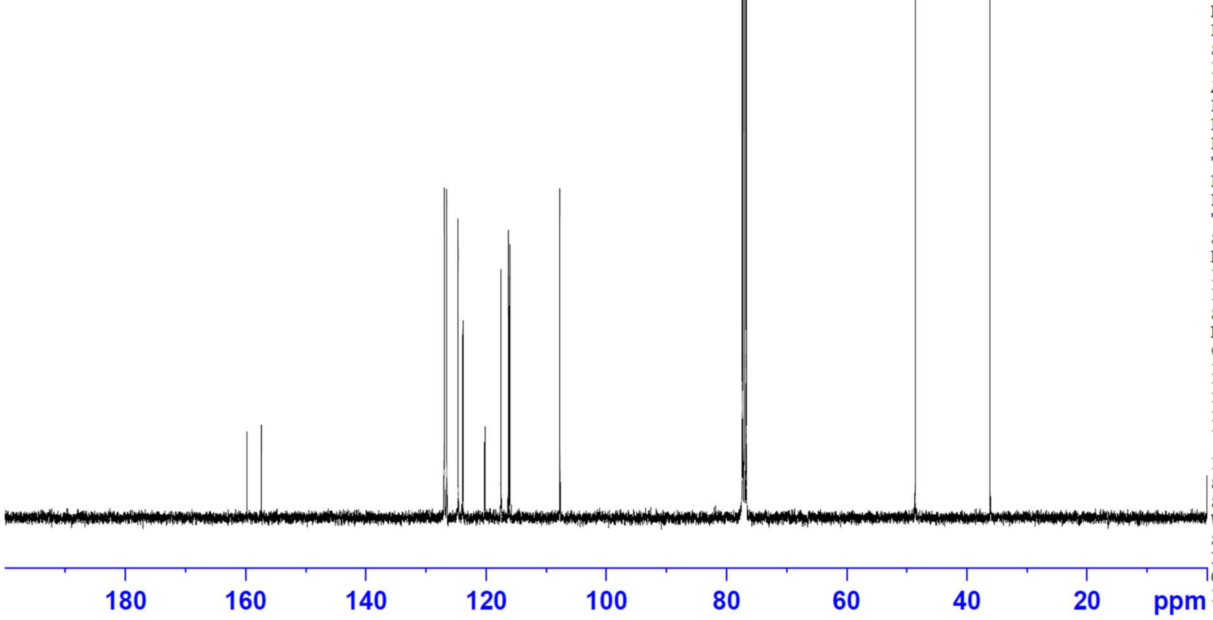

Current Data Parameters
NAME 2022_data
EXPNO 455
PROCNO 1

F2 - Acquisition Parameters
Date_ 20221214
Time 18.14 h
INSTRUM spect
PROBHD Z108618_0899 (
PULPROG zgpg30
TD 65536
SOLVENT CDCl3
NS 1024
DS 4
SWH 24038.461 Hz
FIDRES 0.733596 Hz
AQ 1.3631488 sec
RG 206.42
DW 20.800 usec
DE 6.50 usec
TE 295.3 K
D1 2.00000000 sec
D11 0.03000000 sec
TD0 1
SFO1 100.6228298 MHz
NUC1 13C
P1 10.00 usec
PLW1 70.63899994 W
SFO2 400.1316005 MHz
NUC2 1H
CPDPRG[2 waltz16
PCPD2 90.00 usec
PLW2 15.91600037 W
PLW12 0.38512000 W
PLW13 0.19371000 W

F2 - Processing parameters
SI 32768
SF 100.6127685 MHz
WDW EM
SSB 0
LB 1.00 Hz
GB 0
PC 1.40

431069-VPZ-08-019-1_N A.proton_Ojima

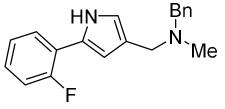

13

^{1}H-NMR (400 MHz, $CDCl_3$)

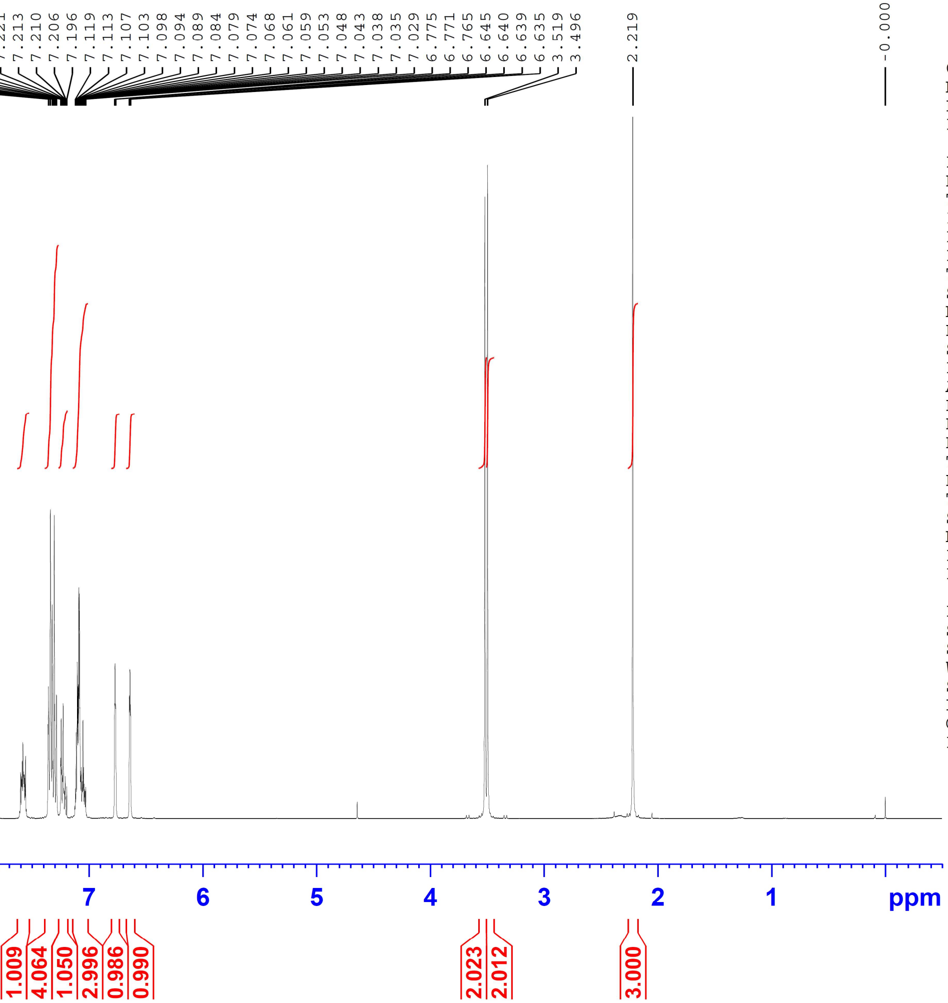

Current Data Parameters
NAME 2022_data
EXPNO 441
PROCNO 1

F2 - Acquisition Parameters
Date_ 20221208
Time 16.36 h
INSTRUM spect
PROBHD Z108618_0899 (
PULPROG zg30
TD 65536
SOLVENT CDCl3
NS 8
DS 2
SWH 8012.820 Hz
FIDRES 0.244532 Hz
AQ 4.0894465 sec
RG 62.83
DW 62.400 usec
DE 6.50 usec
TE 293.9 K
D1 1.00000000 sec
TD0 1
SFO1 400.1324708 MHz
NUC1 1H
P1 14.00 usec
PLW1 15.91600037 W

F2 - Processing parameters
SI 65536
SF 400.1300353 MHz
WDW EM
SSB 0
LB 0.30 Hz
GB 0
PC 1.00

431069-VPZ-08-019-1_N A.carbon13_Ojima

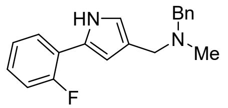

13

^{13}C-NMR (100 MHz, $CDCl_3$)

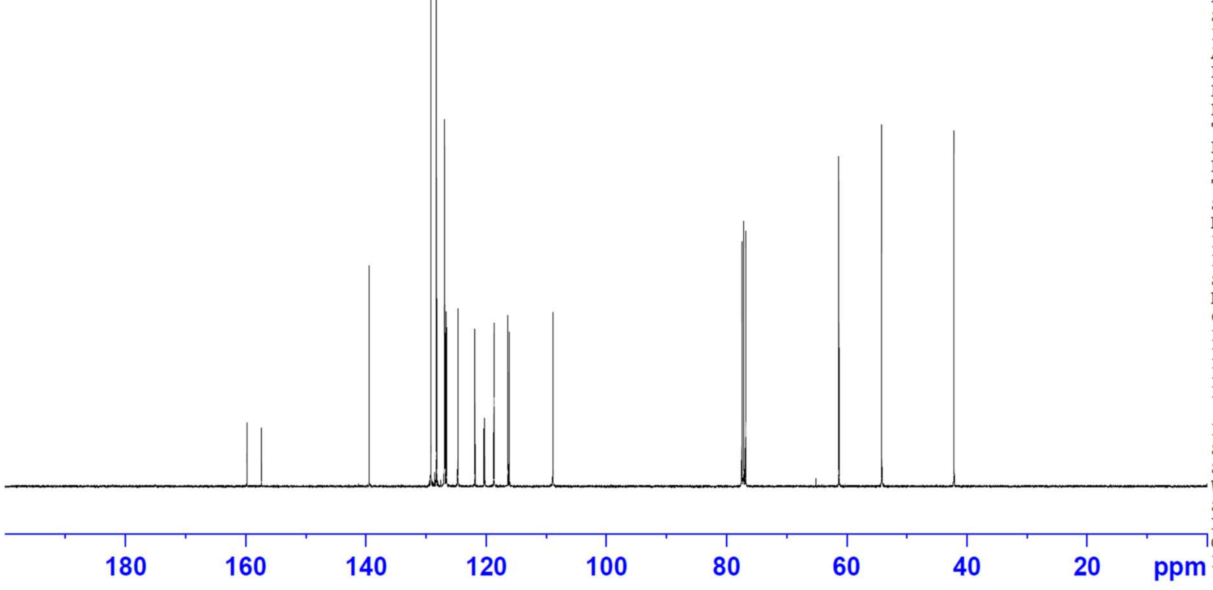

Current Data Parameters
NAME 2022_data
EXPNO 442
PROCNO 1

F2 - Acquisition Parameters
Date_ 20221208
Time 17.36 h
INSTRUM spect
PROBHD Z108618_0899 (
PULPROG zgpg30
TD 65536
SOLVENT CDCl3
NS 1024
DS 4
SWH 24038.461 Hz
FIDRES 0.733596 Hz
AQ 1.3631488 sec
RG 206.42
DW 20.800 usec
DE 6.50 usec
TE 295.3 K
D1 2.00000000 sec
D11 0.03000000 sec
TD0 1
SFO1 100.6228298 MHz
NUC1 13C
P1 10.00 usec
PLW1 70.63899994 W
SFO2 400.1316005 MHz
NUC2 1H
CPDPRG[2 waltz16
PCPD2 90.00 usec
PLW2 15.91600037 W
PLW12 0.38512000 W
PLW13 0.19371000 W

F2 - Processing parameters
SI 32768
SF 100.6127685 MHz
WDW EM
SSB 0
LB 1.00 Hz
GB 0
PC 1.40

431069-VPZ-08-025-1_N A.proton_Ojima

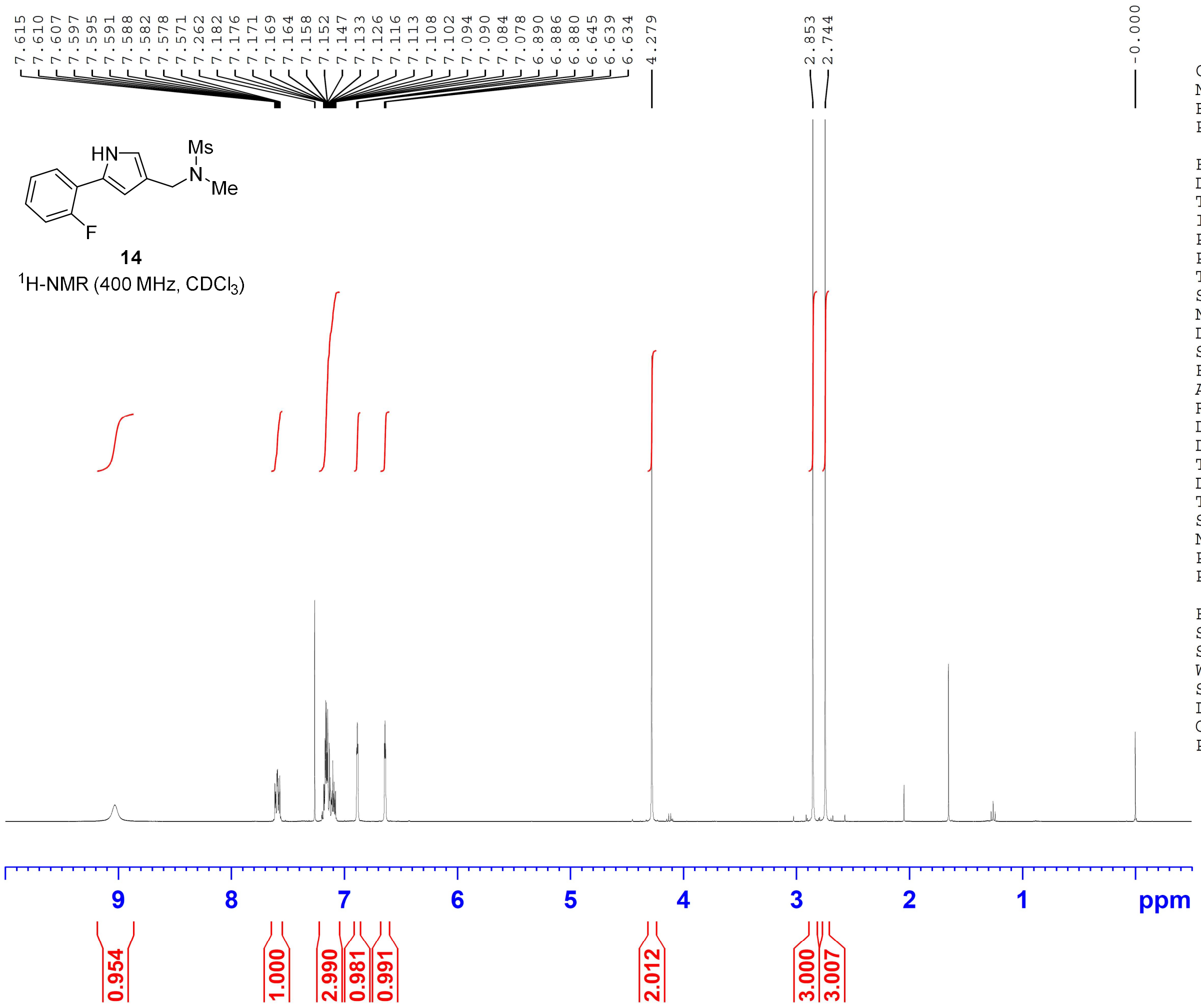

Current Data Parameters
NAME 2022_data
EXPNO 459
PROCNO 1

F2 - Acquisition Parameters
Date_ 20221216
Time 11.34 h
INSTRUM spect
PROBHD Z108618_0899 (
PULPROG zg30
TD 65536
SOLVENT CDCl3
NS 16
DS 2
SWH 8012.820 Hz
FIDRES 0.244532 Hz
AQ 4.0894465 sec
RG 185.95
DW 62.400 usec
DE 6.50 usec
TE 293.0 K
D1 1.00000000 sec
TD0 1
SFO1 400.1324708 MHz
NUC1 1H
P1 14.00 usec
PLW1 15.91600037 W

F2 - Processing parameters
SI 65536
SF 400.1300087 MHz
WDW EM
SSB 0
LB 0.30 Hz
GB 0
PC 1.00

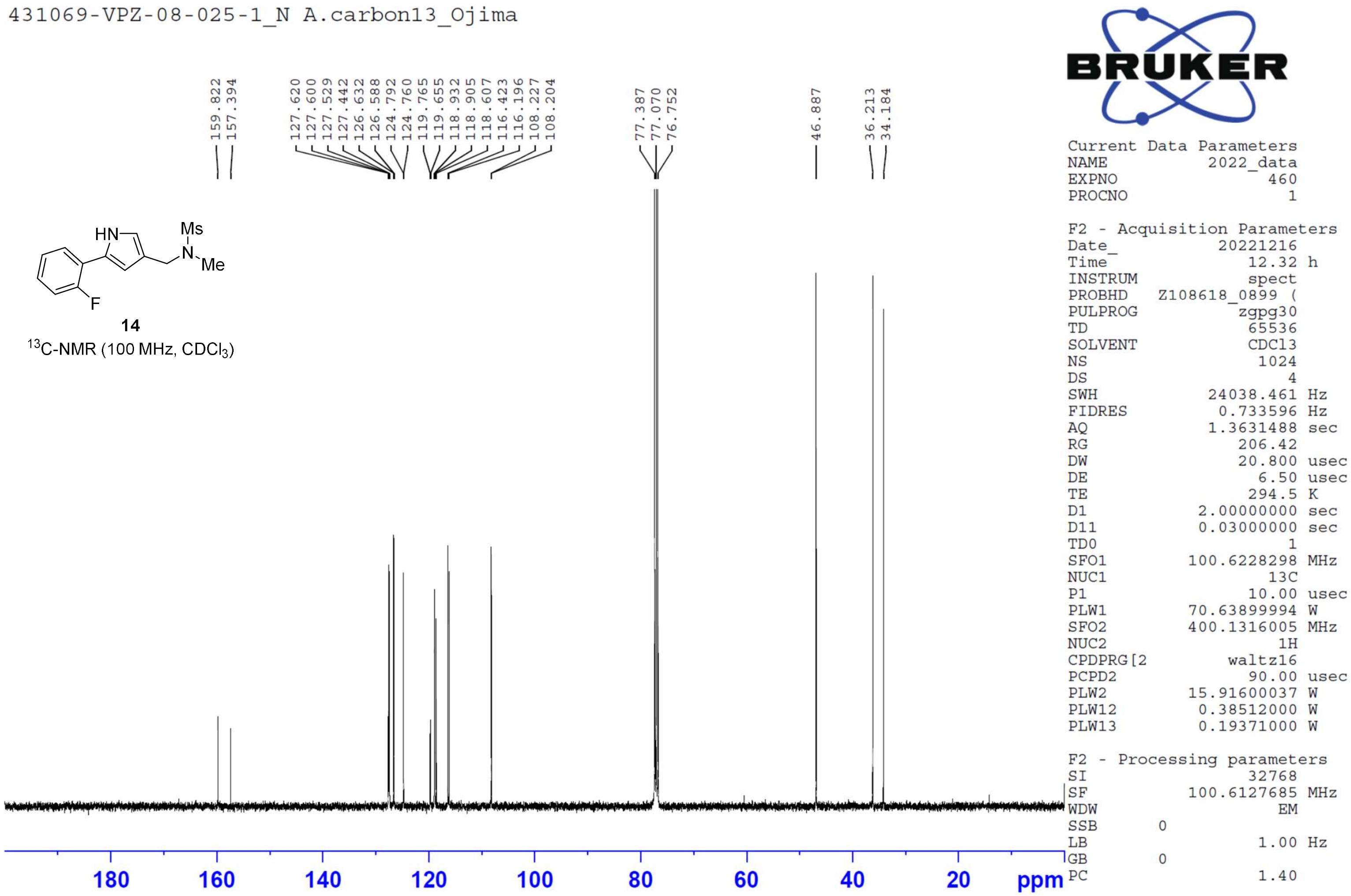

431069-VPZ-08-025-1_N A.carbon13_Ojima
HN
Ms
N
Me
F
14
13C-NMR (100 MHz, CDCl3)
159.822
157.394
127.620
127.600
127.529
127.442
126.632
126.588
124.792
124.760
119.765
119.655
118.932
118.905
118.607
116.423
116.196
108.227
108.204
77.387
77.070
76.752
46.887
36.213
34.184
BRUKER
Current Data Parameters
NAME 2022_data
EXPNO 460
PROCNO 1
F2 - Acquisition Parameters
Date_ 20221216
Time 12.32 h
INSTRUM spect
PROBHD Z108618_0899 (
PULPROG zgpg30
TD 65536
SOLVENT CDCl3
NS 1024
DS 4
SWH 24038.461 Hz
FIDRES 0.733596 Hz
AQ 1.3631488 sec
RG 206.42
DW 20.800 usec
DE 6.50 usec
TE 294.5 K
D1 2.00000000 sec
D11 0.03000000 sec
TD0 1
SFO1 100.6228298 MHz
NUC1 13C
P1 10.00 usec
PLW1 70.63899994 W
SFO2 400.1316005 MHz
NUC2 1H
CPDPRG[2 waltz16
PCPD2 90.00 usec
PLW2 15.91600037 W
PLW12 0.38512000 W
PLW13 0.19371000 W
F2 - Processing parameters
SI 32768
SF 100.6127685 MHz
WDW EM
SSB 0
LB 1.00 Hz
GB 0
PC 1.40
180
160
140
120
100
80
60
40
20
ppm
